# A General Stiffness-Scaling Framework for Accelerating Graph-Theoretical Kinetic Monte Carlo Simulations

**DOI:** 10.1021/acs.jctc.5c01394

**Published:** 2025-11-18

**Authors:** Hector Prats, Weitian Li, Michail Stamatakis

**Affiliations:** Inorganic Chemistry Laboratory, 6396 University of Oxford, South Parks Road, Oxford OX1 3QR, U.K.

## Abstract

Kinetic Monte Carlo (KMC) simulations are a powerful tool for investigating catalytic reaction mechanisms, yet they often become intractably slow when certain fast, quasi-equilibrated reaction channels fire much more frequently than other processes, a problem known as *stiffness*. To overcome this issue, we introduce a new reaction channel-based scaling algorithm that dynamically upscales or downscales the rate constants of quasi-equilibrated channels, ensuring they remain within a user-defined time scale window. In contrast to previous methods that either fully restored original rates after nonequilibrated events or applied one-way downscaling (without the option to increase rates toward their original values), our algorithm adaptively regulates each channel throughout the simulation, and can be applied to both simple and highly complex lattice-based KMC models of catalytic systems. We demonstrate the performance of this method on three representative catalytic systems with adsorbate–adsorbate lateral interactions. First, a reverse water–gas shift (RWGS) model on Ni(111) serves as a benchmark where unscaled simulations are feasible and provide a reference for error analysis. Second, a complex and highly stiff model of dry reforming of methane (DRM) on Pt/HfC–containing 119 reversible channels, multiple site types, and 175 energetic clusters–showcases the algorithm’s robustness across a wide range of time scales and operating conditions (e.g., varying *p*_CH_4__ and *p*_CO_2__). Third, transient simulations of temperature-programmed desorption (TPD) of formate, which entails dissociation, on NiCu single-atom alloys (SAAs) illustrate the method’s ability to adapt to rapid kinetic changes. In all cases, the algorithm is able to significantly accelerate the simulations without introducing substantial error, offering a practical solution to stiffness in KMC studies of catalytic systems. The method is fully integrated into the *Zacros* code (release of the pertinent version pending), making it broadly accessible.

## Graphical abstract

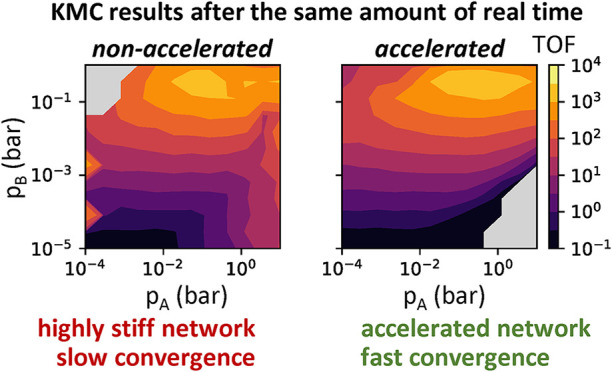

## 1 Introduction

Kinetic Monte Carlo (KMC) simulations are a powerful technique for modeling surface chemistry and studying complex reaction mechanisms in heterogeneous catalysis.^1–6^ By statistically sampling trajectories of the Markovian master equation,^7,8^ KMC connects elementary events such as adsorption, desorption, diffusion, and surface reactions to macroscopic observables like turnover frequencies (TOFs), selectivities, and surface coverages. Beyond these averaged quantities, KMC also reveals dominant reaction pathways and rate-determining steps as a function of operating conditions, even in systems characterized by multiple site types, lateral interactions, and competing mechanisms.^9–18^ These insights are vital for the rational design and optimization of catalytic materials.

A longstanding challenge in KMC simulations is the presence of elementary steps occurring on vastly different time scales in the reaction model. Some fast events, such as certain surface diffusion steps, can occur orders of magnitude more frequently than slower chemical transformations. This issue is known as *stiffness*, adopting terminology from the numerical analysis field, and often leads to prohibitively long simulation times. As a result, significant effort has been devoted to developing acceleration schemes that alleviate this computational bottleneck. All these algorithms are approximate, always introducing some error. The desired case is when this error is small compared to the inherent sampling error in KMC so that it becomes negligible in practice.

An early example of such a scheme is the so-called τ-leap method.^19^ Instead of simulating one event at a time with exact kinetics, the scheme “leaps” forward by a finite time increment (τ), assuming that species populations remain effectively unchanged during that interval. Under this assumption, multiple reaction firings are executed in a single leap, offering substantial computational speedups. However, τ-leap works best for well-mixed systems or coarse-grained lattices where population changes are fairly uniform. It does not readily extend to surface reactions on microscopic lattices, where local site populations can vary drastically over short distances. Another class of algorithms relies on splitting the reaction network into fast and slow processes in advance.^20,21^ The fast subset is integrated with deterministic or Langevin equations while the slow subset is treated stochastically. However, this requires prior knowledge of time scale separation and fails when reaction time scales evolve dynamically or when partitioning is ambiguous.

To address these issues, on-the-fly stiffness scaling methods have emerged. The accelerated superbasin KMC (AS-KMC) algorithm of Chatterjee and Voter^22^ detects groups of rapidly interconverting states (superbasins) and temporarily downscales their internal rates, allowing the simulation to escape from a superbasin much sooner. While AS-KMC has been shown to yield substantial speedups in relatively simple catalytic models, it may become impractical for more realistic models where the total number of states is so large that full sampling of even a single superbasin can be prohibitively expensive.

An important advancement came with *channel-based* scaling, introduced by Dybeck et al.,^23^ which tracks quasi-equilibration at the level of reaction channels (e.g., “H_2_ dissociation on Ni step sites”) rather than individual states. When a channel is identified as fast and equilibrated, its rate constant is downscaled (reduced). After the execution of a nonequilibrated event, the original rates for all scaled channels are restored. This algorithm achieved considerable computational speedups for a relatively simple reaction model where a mean-field approximation (MFA) microkinetic model would have been sufficient. However, for simulations with long initial transients until stationarity is reached, or simulations that never reach stationary conditions, this strategy becomes inefficient as resets can occur too frequently.

Building on this work, Andersen et al.^24^ integrated the Dybeck algorithm into the kmos code^25^ and identified an important limitation in cases where two low-coverage species, produced by different equilibrated channels, must meet to react. In such cases, the algorithm may scale those channels too much, reducing the probability that the two species appear simultaneously at neighboring sites, affecting the accuracy. To mitigate this, Andersen et al. proposed a refinement in which nearest-neighbor environments are included in the definition of a reaction channel (e.g., treating the CO + O reaction channel differently depending on the local configuration). While this correction improves accuracy in some cases, it multiplies the number of distinguishable channels, adding bookkeeping overhead and limiting scalability.

In parallel, “downscaling-only” approaches have been proposed, where once a channel is flagged as fast, its rate constant is only reduced (never restored to a higher value) for the remainder of the simulation. Nuñez et al.^26^ used this strategy in combination with sensitivity analysis, and Hoffmann and Bligaard^27^ applied it to steady-state detection in various CO oxidation models. However, the inability to upscale previously downscaled channels can compromise accuracy in systems with an initial transient period or in temperature-programmed desorption (TPD) simulations, where reaction time scales may change significantly over time.

A more recent contribution by Savva and Stamatakis^28^ proposed an on-the-fly downscaling scheme for well-mixed KMC systems which monitors the error incurred. At regular intervals, the algorithm tests which channels are quasi-equilibrated, generates multiple trial trajectories with successively larger rate reductions, and selects the downscale factor that minimizes an objective function that considers the conflicting goals of high speed-up but also low error. The latter is quantified on the basis of changes in the interarrival times of slow events, and the user can adjust the objective function’s parameters to favor accuracy or computational efficiency. The procedure repeats and can further reduce rate constants as the simulation progresses, although it never restores an already downscaled rate constant to a higher value. Because the framework was developed for well-mixed systems, it is not yet directly applicable to lattice KMC where spatial correlations matter.

Finally, two algorithms by Savara and co-workers, SQERTSS and SQERT-T, introduced the “staggered quasi-equilibrium rank-based throttling” approach for steady-state and transient simulations, respectively. Both variants entail classifying the on-lattice events as fast and frivolous processes (FFPs) versus slow processes (SPs), identifying the fastest rate limiting process (FRP), and then throttling/downscaling the transition rate constants of fast processes to bring them closer to the slow processes, in such a way as to distort the system’s dynamics as little as possible. SQERTSS has been validated on a simplified model of methanol conversion to formaldehyde on CeO_2_(111) and SQERT-T on CO oxidation on RuO_2_(110) and RuO_2_(111), following temperature programs entailing ramps and plateaus in the range of 363 to 453 K. In these works, it was noted that SQERTSS may distort the transient behavior of the simulation, though it is expected to reach the correct final state. SQERT-T was designed to overcome this limitation, but it required the introduction of a fictitious pace-restrictor reaction in the simulation to prevent aggressive downscaling. The rate constant of the pace-restrictor reaction is not known a priori; setting it is subject to heuristics, and improper settings may still lead to loss of accuracy or loss of performance (no significant acceleration). Also, in SQERT-T, slow processes are never downscaled/throttled, while SQERTSS allows this.

In summary, while significant progress has been made, no current method provides a general, efficient, and accurate solution to the stiffness problem in complex KMC models for catalytic systems. Many approaches require manual setup, are unsuitable for evolving kinetics, or have only been validated on simple models.

Here, we introduce a novel channel-based scaling algorithm designed to be robust and broadly applicable to surface chemistry KMC models with many reaction channels, multiple site types, strong lateral interactions, and transient conditions (e.g., temperature-programmed desorption). Our method has been implemented in the *Zacros* code^29^ (release of the pertinent version pending), ensuring ease of adoption by the broader community, and has been benchmarked and validated on three increasingly complex systems.

For these benchmarks, we first evaluate the algorithm on a model of the reverse water–gas shift (RWGS) reaction on Ni(111), which involves fast diffusion events but remains tractable for nonaccelerated simulations. This serves as a benchmark to assess the algorithm’s accuracy across a range of parameter values and quantify any errors introduced by stiffness scaling. Second, we apply the algorithm to a large model of dry-reforming of methane (DRM) on Pt/HfC, comprising (i) a lattice model with 775 lattice sites with three different types; (ii) an energetics model with 175 clusters; and (iii) a reaction model with 119 reversible reaction channels, with time scales spanning many orders of magnitude. We explore hundreds of operating conditions (varying *p*_CH_4__ and *p*_CO_2__) to test the robustness of the algorithm across multiple scenarios. Finally, we test the algorithm in the simulation of a TPD experiment of formate dissociation on a NiCu single-atom alloy (SAA), involving transient kinetics and many fast diffusion channels. This last system tests the algorithm’s adaptability to dynamically evolving kinetics, e.g., channels that transition from fast to slow or vice versa during the temperature ramp. We show that, in all cases, our algorithm can achieve substantial speedups while preserving accuracy, thereby enabling KMC simulations of complex catalytic systems previously out of reach due to stiffness.

## 2 Background on the KMC Method

The KMC method^1,30–32^ provides a way to simulate the long-time evolution of catalytic systems by “jumping” among distinct potential-energy basins, each identified as a KMC state ω. Because a system remains in any given basin for a relatively long period compared to atomic vibration time scales, it loses memory of how it arrived there. This justifies treating the transitions among states as *Markovian*—i.e., each transition probability depends only on the current state and the candidate state to be reached next, but not on the previous history.

Each elementary event that carries the system from one state ω to a different state ω′ is associated with a rate constant *k*_ω→ω′_. The latter can be obtained from transition-state theory (TST), which depends on the free-energy barrier between ω and the transition state leading to ω′. In simple Arrhenius form, one often writes

$$k_{\omega \to \omega^{\prime }}=\kappa \frac{k_{B}T}{h}\frac{Q^{†}}{Q}\exp\left(-\frac{E_{\omega \to \omega \prime }^{†}}{k_{B}T}\right)$$ 1

where κ is a transmission coefficient, *k*_B_ is Boltzmann’s constant, *T* is the temperature, *h* is Planck’s constant, *Q* and *Q* ^†^ denote quasi-partition functions accounting for the vibrations, rotations and translations (if applicable) for the initial and transition states, respectively, and *E*_ω→ω′_^†^ is the activation energy.

When the system is in state ω, several possible elementary events may occur, each with its own rate constant. The *total* escape rate out of ω is *k*_tot_ = ∑_ω′_ *k*_ω→ω′_. For time-independent rate constants, the waiting time τ before the system leaves state ω follows an exponential distribution with parameter *k*_tot_

2 $$p\left(\tau \right)=k_{\mathrm{tot}}\exp\left(-k_{\mathrm{tot}}\tau \right)$$

which arises naturally from the Poisson process that underlies Markovian transitions. The probability that the system has left ω by time τ is obtained from the cumulative distribution function:

3 $$P\left(\tau_{\mathrm{escape}}\leq \tau \right)=1-\exp\left(-k_{\mathrm{tot}}\tau \right)$$

Because the *escape time τ*_ω→ω′_ (also referred to as occurrence or *waiting time*)^30^ for each individual event ω→ω′ is also exponentially distributed with rate *k*_ω→ω′_, one can generate random escape times in a simulation using the inverse transformation method.^30^ Let *u*_1_∈(0,1) be a uniform random number. Then

$$\tau_{\omega \to \omega^{\prime }}=-\frac{1}{k_{\omega \to \omega^{\prime }}}\ln\left(1-u_{1}\right)$$ 4

The first event to occur is the one with the minimum of all τ_ω→ω′_ values, also known as the most imminent event. Then, the simulation time is advanced by τ_adv_ = τ_ω→ω′_, where τ_ω→ω′_ is the minimum escape time. This is known as the first reaction method (FRM),^30,33^ whereby a random occurrence time calculated from eq 4 is assigned to each possible event (for a given lattice state), and the one with the smallest time is selected.

Equivalently, one can sample a single τ_adv_ directly from an exponential distribution with parameter *k*_tot_

5 $$\tau_{\mathrm{adv}}=-\frac{1}{k_{\mathrm{tot}}}\ln\left(1-u_{1}\right)$$

and then choose which particular event occurs by mapping a second uniformly distributed random number *u*_2_ onto an integer *q*∈{1,···,*M*} to select among all possible transitions proportionally to their rates, i.e.

6 $$\sum_{m=1}^{q-1}k_{\omega \to \omega_{m}^{\prime }}<u_{2}k_{\mathrm{tot}}\leq \sum_{m=1}^{q}k_{\omega \to \omega_{m}^{\prime }}$$

This is known as the direct method (DM),^30^ in which the single next event and its occurrence time are directly sampled from the above eqs 5 and 6.

In practice, a significant portion of the computational cost in a KMC simulation arises from deciding which event will happen and from managing the data structures that track possible events. Two main algorithmic families exist: null-event algorithms, where some selected events prove nonrealizable and therefore get “rejected,” and rejection-free algorithms, where every event chosen is guaranteed to be executed. Both are formally exact and yield statistically equivalent results, but their efficiency can differ substantially. Over time, rejection-free methods have become the standard for heterogeneous catalysis simulations, typically in one of two variants discussed above, FRM or DM. Both approaches are statistically identical but rely on different data structures for speed. Detailed discussions on KMC algorithms can be found elsewhere.^30–32^

A KMC simulation begins by defining the lattice structure, reaction mechanism, and energetic parameters (including lateral interactions), along with operating conditions such as pressure, temperature, and gas-phase composition. With these inputs specified, the simulation starts from an initial lattice configuration (frequently taken as an empty lattice). Then, the algorithm identifies all possible elementary events and compiles them in a queue. In the FRM variant, the simulation continues by selecting and executing the most imminent event, updating the lattice configuration and time clock, and modifying the queue accordingly by removing invalidated events and adding any newly enabled ones. This cycle is repeated at each simulation step, and the simulation continues until a stopping condition is met (e.g., reaching a user-specified maximum time). The resulting trajectory data can then be analyzed to extract catalytic performance metrics, coverages, or reaction pathways. A typical flowchart of the FRM is illustrated in Figure 1 (procedures in orange and blue boxes), as it is the one implemented in the *Zacros* code, the software that we use here.

**Figure 1:**
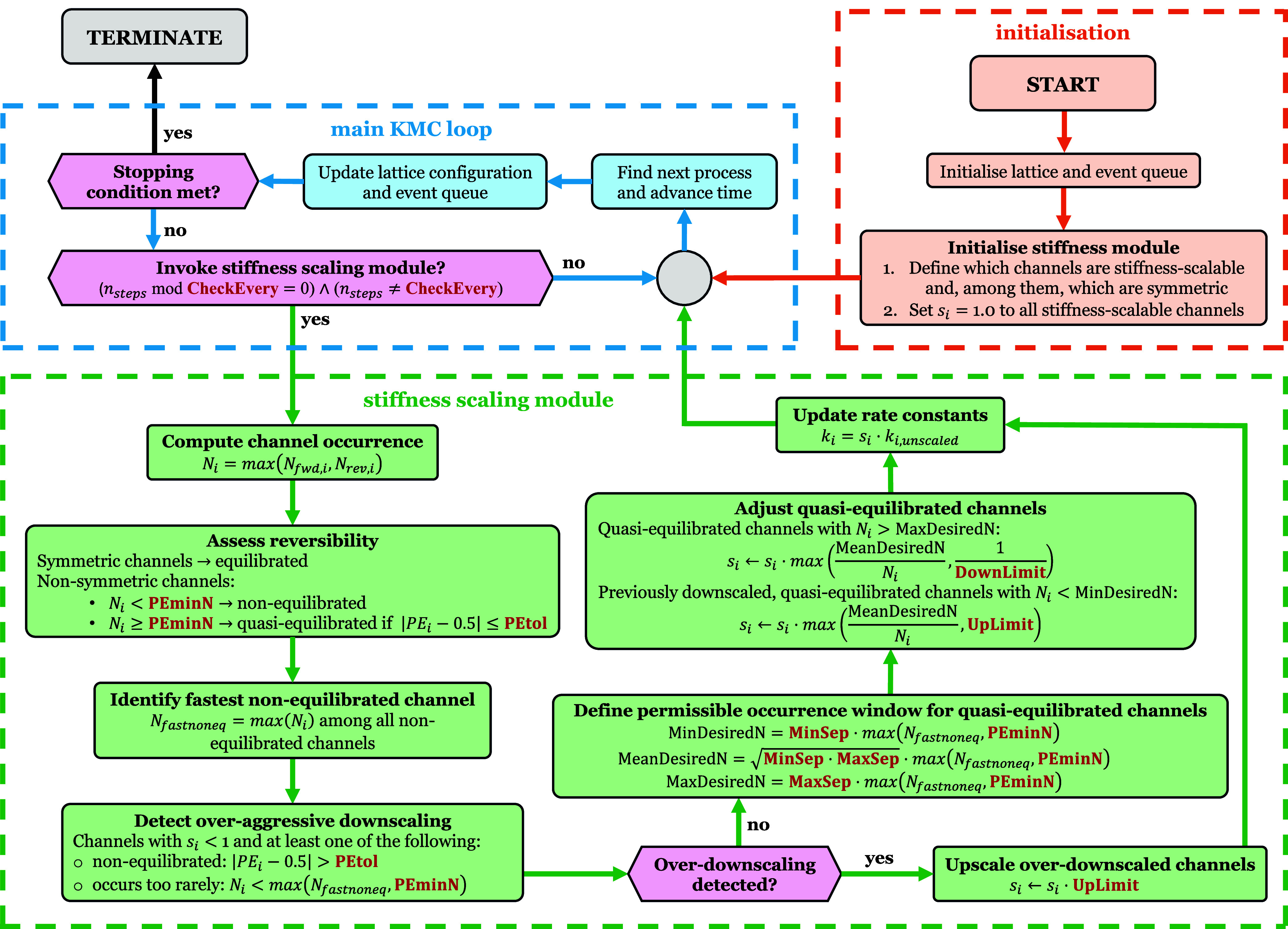
Flowchart illustrating the FRM version of the standard KMC algorithm and the additional steps corresponding to the stiffness scaling module. Keywords in red correspond to user-defined parameters. Orange, blue and green colors are used to distinguish between the initialization, the main KMC loop, and the stiffness scaling module steps, respectively.

Formally, KMC can be seen as a stochastic realization of the Markovian master equation^7^

$$\frac{{dp}_{\omega }\left(t\right)}{dt}=\underset{\omega^{\prime }}{\sum }k_{\omega^{\prime }\to \omega }\;p_{\omega^{\prime }}\left(t\right)-\underset{\omega^{\prime }\;}{\sum }k_{\omega \to \omega^{\prime }}\;p_{\omega }\left(t\right)$$ 7

where *p*_ω_(*t*) is the probability that the system occupies state ω at time *t*. At steady-state, the net flux into each state equals the net flux out

$$\underset{\omega^{\prime }\;}{\sum }k_{\omega^{\prime }\to \omega }\;p_{\omega^{\prime }}^{\mathrm{ss}}=\underset{\omega^{\prime }\;}{\sum }k_{\omega \to \omega^{\prime }}\;p_{\omega }^{\mathrm{ss}}$$ 8

At thermodynamic equilibrium, the constraint of detailed balance applies

$$\frac{k_{\omega \to \omega^{\prime }}}{k_{\omega^{\prime }\to \omega }}=\exp\left[-\frac{\left(\varepsilon_{\omega^{\prime }}-\varepsilon_{\omega }\right)}{k_{B}T}\right]$$ 9

where ε_ω_ and ε_ω′_ are the free energies associated with states ω and ω′, respectively.

## 3 Novel Stiffness Scaling Algorithm

### 3.1 Overview of the Algorithm

Building on the channel-based approach introduced by Dybeck et al.,^23^ we have developed a more general stiffness scaling algorithm that overcomes key limitations of previous methods. While Dybeck’s procedure upscales the rate constants of *all* reaction channels back to their original values whenever a nonequilibrated event occurs—a strategy that becomes impractical in large, complex reaction networks—the more efficient “downscaling-only” methods^26,27^ can lead to inaccuracies when processes that start off fast eventually slow down, yet remain artificially suppressed. Our algorithm overcomes these issues by allowing *both* upscaling and downscaling in a controlled manner, guided by user-defined thresholds and protective measures that prevent overaggressive rate adjustments. Figure 1 shows a flowchart of the procedure.

A central idea of this algorithm is to periodically pause the simulation after a prescribed number of KMC events, determined by the user-defined parameter **CheckEvery**, and assess each reaction channel’s statistics (e.g., forward and reverse occurrence counts) in order to (i) identify whether the time scales of certain channels are too fast or too slow relative to the rest of the network, and (ii) detect whether any downscaled channel has been suppressed too aggressively. Moreover, this algorithm limits how drastically rate constants can be altered at each **CheckEvery** interval via the user-defined **DownLimit** and **UpLimit** parameters to prevent large swings that may distort system kinetics.

At the start of the KMC simulation, each reaction channel *i* is assigned a stiffness coefficient *s*_*i*_ = 1. After every **CheckEvery** steps except after the first interval (which is only used to obtain reaction occurrence statistics), the algorithm updates these coefficients based on the channel occurrence counts of the last interval. The new rate constant *k*_*i*_ of channel *i* is then calculated as

10 $$k_{i}=s_{i}\cdot k_{i,\mathrm{unscaled}}$$

where *k*_i,unscaled_ is the original (unscaled) rate constant, typically obtained from TST. Note that the stiffness coefficients are constrained to lie within the interval [0, 1]. Rate constants are updated only at these periodic checkpoints (integer multiples of **CheckEvery** steps), which keeps the overhead of the scaling procedure very low.

#### 3.1.1 Partial Equilibrium Ratio and Reversibility

As in previous algorithms, reaction channels are classified as either quasi-equilibrated or nonquasi-equilibrated (for brevity we may, in the following, use the terms “equilibrated” and “non-equilibrated”, respectively). This classification is done by evaluating each channel’s partial equilibrium (PE) ratio

11 $${\mathrm{PE}}_{i}=\frac{N_{\mathrm{fwd},i}}{N_{\mathrm{fwd},i}+N_{\mathrm{rev},i}}$$

where *N*_fwd,i_ and *N*_rev,*i*_ are the forward and reverse occurrence counts of channel *i* during the last **CheckEvery** steps. We define the *occurrence count* of channel *i* as the maximum between the forward and reverse directions

12 $$N_{i}=\max\left(N_{\mathrm{fwd},i},N_{\mathrm{rev},i}\right)$$

Channels with *N*_*i*_ > 0 are referred to as *active* (i.e., they have occurred at least once during the last **CheckEvery** steps), while those with *N*_*i*_ = 0 are referred to as *inactive*. A channel is labeled as *quasi-equilibrated* if

13 $$\left|{\mathrm{PE}}_{i}-0.5\right|\leq \mathrm{PEtol}$$

where **PEtol** is a user-defined tolerance. Two main exceptions apply. First, symmetric channels (e.g., diffusion between equivalent sites) are always treated as quasi-equilibrated to avoid redundant computations for processes that have identical forward and reverse directions. Second, if a channel has occurred too few times (i.e., *N*_*i*_ < **PEminN**, where **PEminN** is another user-defined parameter), it cannot be reliably classified via the PE ratio and is thus labeled nonequilibrated by default. This prevents mislabeling poorly sampled events as equilibrated simply because they happened to occur a very similar number of times in the forward and reverse directions.

#### 3.1.2 Overaggressive Downscaling

The algorithm continues by checking whether any previously downscaled channel is either (i) no longer equilibrated or (ii) occurring too rarely (Figure 1). Specifically, this means that a channel with *s*_*i*_ < 1 has been downscaled too much if it fails the equilibrium criterion (eq 13), or has

$$N_{i}<\max\left(N_{\mathrm{fastnoneq}},\mathrm{PE}\min N\right)$$ 14

where *N*_fastnoneq_ is the occurrence count of the *fastest nonequilibrated channel*, i.e., the channel with the highest occurrence count among all nonequilibrated channels. Such situations can arise during the initial transient periods before stationarity is reached (where a channel starts off fast but subsequently slows) or under transient kinetic experiments (e.g., temperature-programmed desorption). If the algorithm detects that one or more channels have been downscaled too much, their stiffness coefficients are increased by a factor of **UpLimit** to correct for excessive suppression, while ensuring, however, that *s*_*i*_ does not exceed 1. Further scaling actions for other channels are then skipped in the current cycle to prevent making additional decisions based on potentially skewed event statistics.

#### 3.1.3 Adjusting Time Scales of Quasi-Equilibrated Channels

If no overaggressive downscaling is detected, the algorithm checks which channels require rate adjustments (Figure 1). A key goal of the algorithm is to maintain all quasi-equilibrated channels within a defined time scale window. This range is determined by the user-specified parameters **MinSep** and **MaxSep**, which define the lower and upper thresholds, and the corresponding target values are determined as follows

$$\mathrm{MinDesiredN}=\mathrm{MinSep}\cdot \max\left(N_{\mathrm{fastnoneq}},\mathrm{PE}\min N\right)$$ 15

$$\mathrm{MeanDesiredN}=\sqrt{\mathrm{MinSep}\cdot \mathrm{MaxSep}}\cdot \max\left(N_{\mathrm{fastnoneq}},\mathrm{PE}\min N\right)$$ 16

$$\mathrm{MaxDesiredN}=\mathrm{MaxSep}\cdot \max\left(N_{\mathrm{fastnoneq}},\mathrm{PE}\min N\right)$$ 17

which translate desired time scale ranges into thresholds on observed occurrence counts for fast, quasi-equilibrated channels. Those channels with *N*_*i*_ > MaxDesiredN are *too fast* and thus downscaled by

18 $$s_{i}\leftarrow s_{i}\cdot \max\left(\frac{\mathrm{MeanDesiredN}}{N_{i}},\frac{1}{\mathrm{DownLimit}}\right)$$

while previously downscaled channels with *N*_*i*_ < MinDesiredN are *too slow* and thus upscaled by

19 $$s_{i}\leftarrow s_{i}\cdot \min\left(\frac{\mathrm{MeanDesiredN}}{N_{i}},\mathrm{UpLimit}\right)$$

In both situations, adjustments are constrained by **DownLimit** and **UpLimit** to prevent overaggressive downscaling or upscaling that can distort the simulation dynamics, and always ensuring that *s*_*i*_ does not exceed 1.

#### 3.1.4 Resuming the Simulation

Finally, the rate constants of all reaction channels are updated based on the new stiffness coefficients, and the simulation continues for another block of **CheckEvery** steps. If the parameter choices are well tuned, and the simulation does not involve transient conditions, the system should settle into a regime where quasi-equilibrated channels no longer require further scaling. Their stiffness coefficients will stabilize or fluctuate only slightly, in the same way that coverages or event frequencies do at steady-state.

### 3.2 Parameter Reduction and Default Values

Although the stiffness-scaling algorithm described above is designed to be robust and flexible, it introduces seven user-defined parameters that can make the method difficult to learn and optimize. To simplify usage while preserving flexibility, we have implemented a parameter-reduction strategy in which only a few parameters are directly specified by the user, while the remaining ones are assigned default values through algebraic relationships that align with the logic of the algorithm.

These seven parameters can be grouped in three categories according to their function in the algorithm. The first category involves **PEtol**, **PEminN**, and **CheckEvery** parameters, which deal with sampling and classification. **PEtol** determines how strictly the method classifies a channel as quasi-equilibrated (eq 13). Tighter partial equilibrium tolerances improve accuracy but also restrict the number of channels that qualify as quasi-equilibrated (considering also the stochastic fluctuations in the number of event occurrences), thereby limiting the speedup. Very loose tolerances have the opposite effect: they can mis-label nonequilibrated channels as equilibrated, leading to overaggressive downscaling.

Closely related is **PEminN**, which protects against misclassification of poorly sampled channels. Consider a slow channel that occurs only once in each direction during a given **CheckEvery** interval, i.e., *N*_fwd,*i*_ = *N*_rev,*i*_ = 1. This channel would technically satisfy the PE criterion, but only by chance. Hence, the algorithm must impose a minimum occurrence count *N*_*i*_ before it considers a channel eligible for equilibrium assessment, and this threshold should scale with **PEtol**. One can show (see proof in Appendix A) that a channel with a minimum occurrence of

20 $$N_{i}>\frac{\mathrm{PEtol}+0.5}{2\times \mathrm{PEtol}}$$

will always classify as equilibrated if |*N*_fwd,*i*_ – *N*_rev,*i*_ | ≤ 1, and could only be classified as nonequilibrated when |*N*_fwd*,i*_ – *N*_rev,*i*_ | > 1, depending on its PE ratio. Eq 20 therefore sets a lower bound on occurrence counts required for a reliable classification. This condition can be incorporated by defining **PEminN** as

$$\mathrm{PE}\min N=a_{1}\times \frac{\mathrm{PEtol}+0.5}{2\times \mathrm{PEtol}}$$ 21

where *a*_1_ is a scaling factor that must be greater than 1 to guarantee that eq 20 is satisfied under all circumstances. As shown in the next section, *a*_1_ = 1.5 offers a good performance.

The last parameter from this first category, **CheckEvery**, determines the length of the sampling window. This window must be long enough to ensure that if a fast, quasi-equilibrated channel exists, its number of occurrences would surpass the **MaxSep** × **PEminN** threshold in one direction; otherwise, the algorithm will never mark it as *too fast* (eq 17) and no downscaling will occur. Because, on average, only half of the occurrences of fast, quasi-equilibrated channels are on the forward direction and the other half on the reverse, the shortest possible window should contain 2 × **MaxSep** × **PEminN** steps. To take this into account, a suitable default for **CheckEvery** is

$$\mathrm{CheckEvery}=a_{2}\times \mathrm{MaxSep}\times \mathrm{PE}\min N$$ 22

where *a*_2_ is another scaling factor that must be greater than 2. Low values of *a*_2_ (i.e., narrow **CheckEvery** windows) result in more frequent stiffness coefficient updates, making the algorithm responsive but at the cost of noisier statistics, so quasi-equilibrated channels may be (temporarily) labeled as nonequilibrated, suppressing the acceleration. Moreover, in systems with several fast channels, an overly small *a*_2_ can prevent any of them from accumulating more than MaxDesiredN events in the sampling window (eq 17), again limiting acceleration or even completely preventing it. Conversely, very large *a*_2_ values imply a very long sampling window, which is safe at steady-state but risky during transient periods: outdated statistics may cause overaggressive downscaling of channels that start fast but subsequently slow down. While the algorithm can rescale a mistakenly downscaled channel (Section 3.1.2), an excessively large *a*_2_value can permanently distort the dynamics of the system. In Section 4 we show that the best performance is obtained for a value of *a*_2_ similar to the number of fast channels in the reaction model.

The second category involves **MinSep** and **MaxSep**, which control the target time scale separation for fast, quasi-equilibrated events. Increasing the required time-scale separation steadily lowers both acceleration and error. In the limit of very large separation, no channel is ever down-scaled. Users can always lower this separation to push the algorithm toward more aggressive acceleration, but they should verify that the resulting increase in error remains acceptable for their analysis goals. A reasonable choice is to make **MaxSep** a multiple of **MinSep**:

$$\mathrm{MaxSep}=a_{3}\times \mathrm{MinSep}$$ 23

where *a*_3_ is another scaling factor that must always be greater than 1 to ensure that **MaxSep** > **MinSep**. A good default is *a*_3_ = 2, as we shall see in Section 4.

The third and last category involves **DownLimit** and **UpLimit**, which cap how much a stiffness coefficient can be reduced or increased in a single adjustment. Too conservative values can slow down or even prevent driving the quasi-equilibrated channels into the target window, while too loose values can trigger oscillations due to overaggressive downscaling or upscaling, especially during the initial transient period. To keep the focus on one of these parameters, **UpLimit** can default to a multiple of **DownLimit**:

$$\mathrm{UpLimit}=a_{4}\times \mathrm{DownLimit}$$ 24

where the scaling factor *a*_4_ must be ≥1/**DownLimit** to ensure that **UpLimit** is always ≥1. A good default is *a*_4_ = 1, to ensure that channels are upscaled and downscaled at a similar level of aggressiveness.

In summary, we propose that three out of the seven parameters are directly specified (**PEtol**, **MinSep**, and **DownLimit**), while the remaining four are automatically calculated from these three using simple algebraic expressions involving scaling factors, which help prevent these parameters from having incompatible values (eqs 21–24). We also find that a reasonable compromise between aggressiveness and caution can be obtained with **PEtol** = 0.02, **MinSep** = 500, **DownLimit** = 5, and determining the remaining four parameters with eqs 21–24 using the following scaling factor values: *a*_1_ = 1.5 for **PEminN**, *a*_2_ similar to the number of fast channels for **CheckEvery** (e.g., 5 in simple reaction models and 10–30 in larger reaction networks), *a*_3_ = 2 for **MaxSep**, and *a*_4_ = 1 for **UpLimit**. Users can always lower **MinSep** to push the algorithm toward more aggressive acceleration, but they should anticipate the accompanying increase in the error and verify that it remains acceptable for their analysis goals. Advanced users can ignore these default values by explicitly defining the values of the 7 scaling parameters to better accommodate unusual regimes. For instance, extremely stiff networks might require a higher **DownLimit** value than the default one.

## 4 Results and Discussion

We proceed to discuss our tests of the algorithm’s performance on three representative systems, all derived from previously published KMC models with some modifications in two of them. Full details of the three KMC models, including input and output files, are available in a public repository (see Data Availability section).

### 4.1 Performance on a Minimal Benchmark: RWGS on Ni(111)

We begin this section by analyzing the performance of the algorithm on a simple model of the RWGS reaction (CO_2_ + H_2_ → CO + H_2_O) model on Ni(111) which serves as a benchmark in which unscaled KMC results are tractable for error quantification. This KMC model is derived from the work by Lozano et al.^34^ Here, we use a simplified mechanism with a lower number of reaction channels and artificially increase the energy barriers for the mechanism’s four diffusion channels by 0.35 eV. Without this change, nonaccelerated simulations would be prohibitively slow, making error quantification impossible. The mechanism adopted here is depicted in Figure 2A, and the updated energy barriers are marked with an asterisk. The lattice model is a 10 × 10 hexagonal periodic lattice where all sites are equivalent. All simulations were run for 2.5·10^8^ KMC events at the following operating conditions: 800 K, 0.4 bar of CO_2_, and 1.6 bar of H_2_.

**Figure 2:**
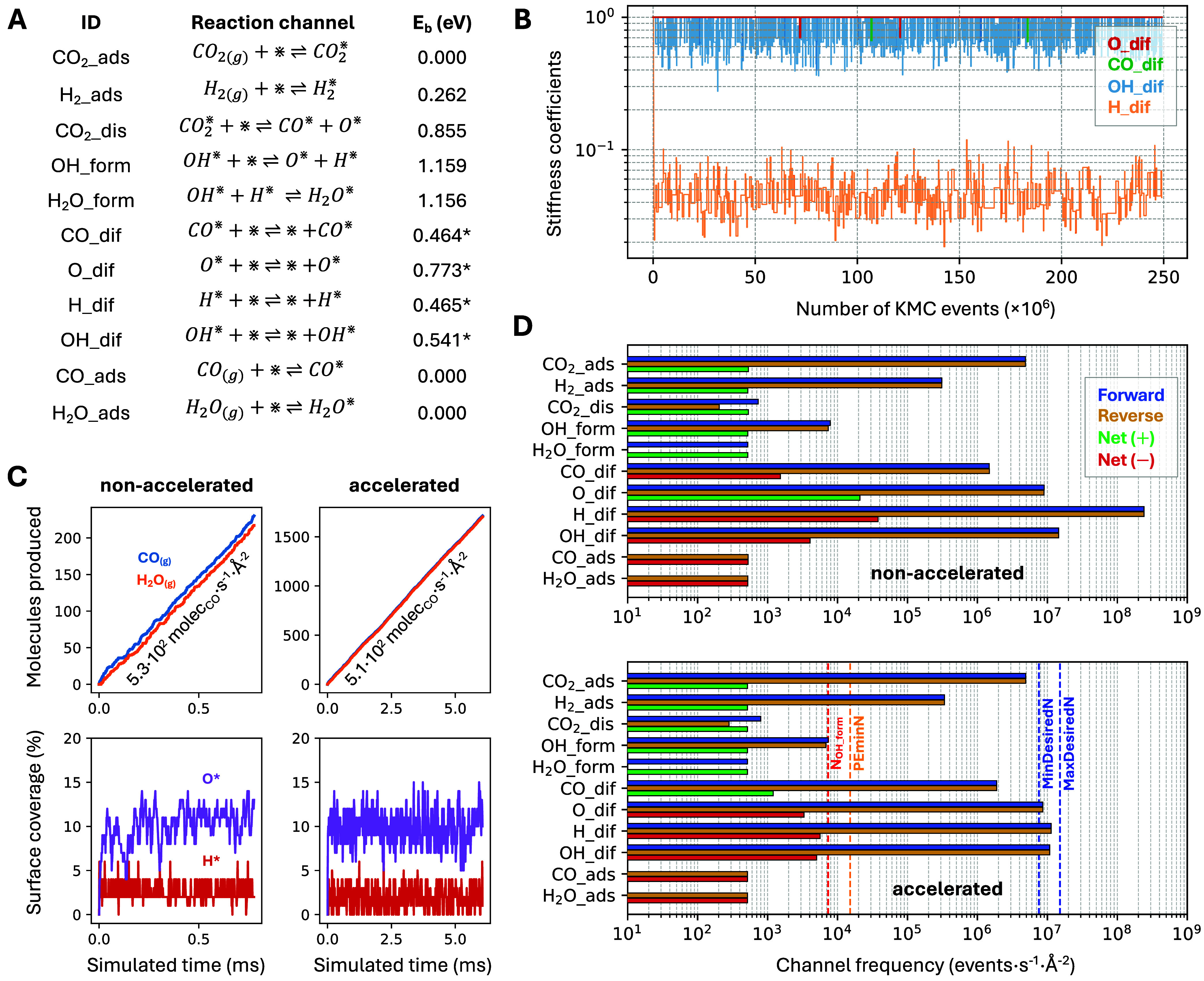
(A) Reaction channels included in the RWGS on Ni(111) model (800 K, *p*_CO2_ = 0.4 bar, and *p*_H2_ = 1.6 bar) with energy barriers. Asterisks indicate values increased by 0.35 eV relative to ref 34. (B) Evolution of stiffness-scaling coefficients during the accelerated KMC run. (C) Cumulative gas-phase production of CO and H_2_O (top) and surface coverages of H and O (bottom) for the nonaccelerated (left) and accelerated (right) simulations. (D) Forward, reverse and net channel frequencies for the nonaccelerated (top) and accelerated (bottom) simulations; vertical dashed lines denote the frequencies corresponding to the fastest nonequilibrated channel (red), **PEminN** (blue), and range of permissible time scales for the fast, quasi-equilibrated channels determined by **MinSep** and **MaxSep** (blue). Channel frequencies have been only computed for the second half of the simulated time, to account for the initial transient period. The parameters used for the accelerated simulations are **PEtol** = 0.02, **MinSep** = 500, **DownLimit** = 5, **PEminN** = 20 (*a*_1_ = 1.5), **CheckEvery** = 10^5^ (*a*_2_ = 5), **MaxSep** = 1000 (*a*_3_ = 2), and **UpLimit** = 5 (*a*_4_ = 1). All simulations were run for 2.5·10^8^ KMC steps.

Figure 2B shows the evolution of the stiffness coefficients for an accelerated simulation as a function of the cumulative number of KMC events, using the default values recommended in the previous section. The coefficients of the H and OH diffusion channels (H_dif and OH_dif) drop rapidly from 1 to approximately 0.05 and 0.8, respectively, and then fluctuate around these values, while the other channels are not downscaled. This result is in line with the ranking of channel frequencies for the nonaccelerated simulations (Figure 2D, top), where H diffusion is by far the fastest channel, followed by OH diffusion. The downscaling of these two channels results in a significant acceleration, increasing the final simulated time from ∼0.7 to ∼6 ms for the given number of KMC steps (Figure 2C).

The accelerated and nonaccelerated simulations yield very similar values for the production rates and surface coverages over the entire simulation window (Figure 2C). Section 4.2 shows that, for the parameters used here, the difference in CO TOF between the accelerated and nonaccelerated simulations remains well inside the ± 2σ confidence interval of the nonaccelerated simulations when running several statistically independent replicas. Importantly, the algorithm keeps the average frequencies of the scaled diffusion channels (i.e., H_dif and OH_dif) within the user-defined window delimited by MinDesiredN and MaxDesiredN (Figure 2D, bottom), confirming that the prescribed time-scale separation is respected throughout the run.

### 4.2 Parameter Sensitivity Analysis on RWGS Model

#### 4.2.1 Influence of Primary Scaling Parameters

To quantify how the user-defined parameters affect accuracy and speedup we focus on the four parameters expected to have the greatest influence: **MinSep**, **PEtol**, **PEminN** (via *a*_1_) and **CheckEvery** (via *a*_2_). We tested three representative values for each of these parameters and considered all possible combinations (Figure 3), leading to 3^4^ = 81 distinct parameter sets. For every set we ran five statistically independent replicas (i.e., different random seed), each comprising 2.5·10^8^ KMC events. An additional set of 5 replicas for the nonaccelerated simulation was also run which serves as a reference.

**Figure 3:**
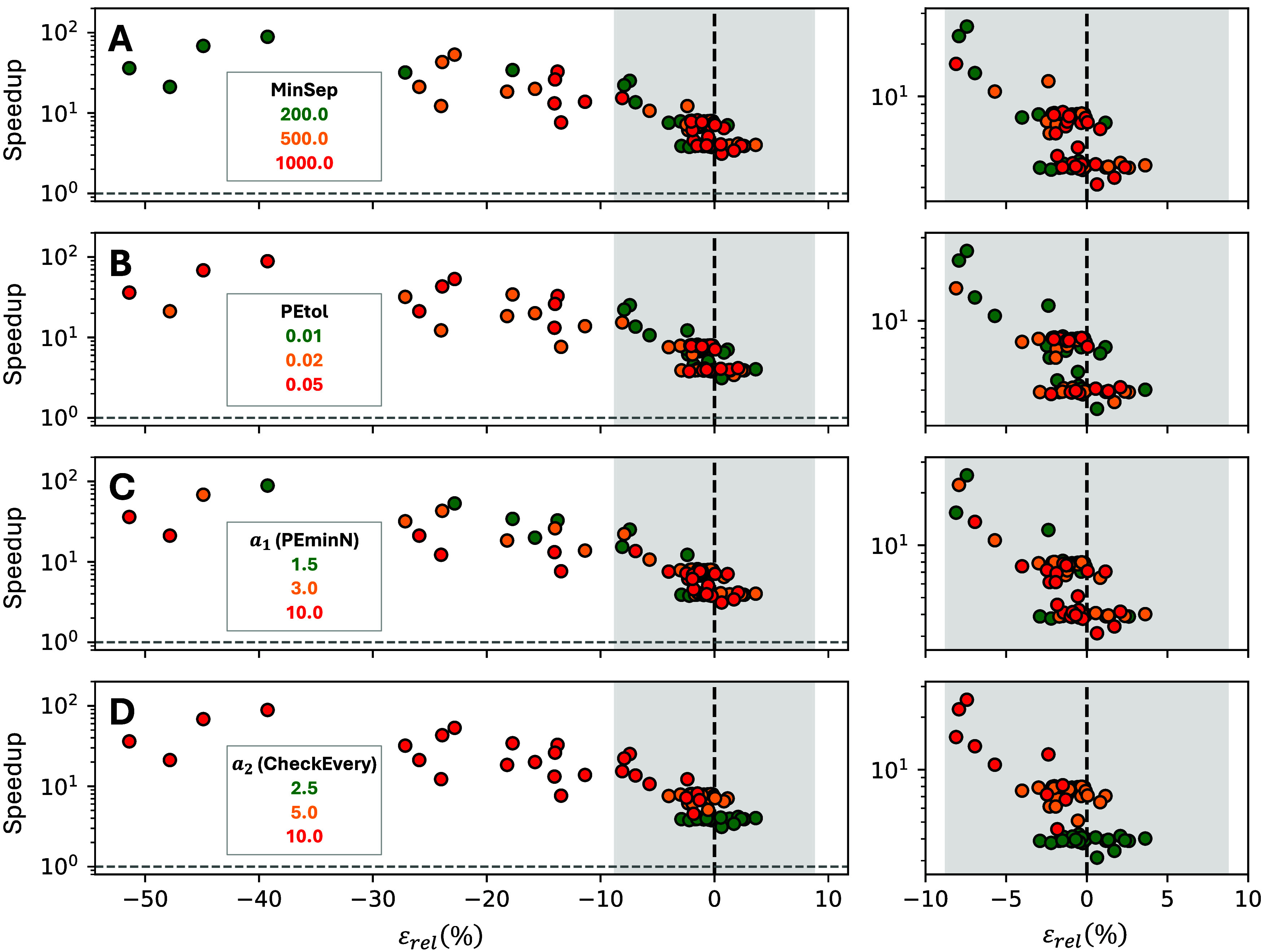
Influence of (A) **MinSep**, (B) **PEtol**, (C) **PEminN** (via scaling factor *a*_1_) and (D) **CheckEvery** (via scaling factor *a*_2_) on the trade-off between speedup (*y*-axis) and signed relative error (*x*-axis) for the accelerated algorithm on the RWGS on Ni(111) model (800 K, *p*_CO2_ = 0.4 bar, and *p*_H2_ = 1.6 bar). The speedup and error are defined according to eqs 25 and 26. The right-side subplots provide a zoomed-in view to emphasize performance in low-error regimes. Each data point is obtained by averaging over five independent replicas with different random seeds. The light-gray vertical band indicates ±2σ of the nonaccelerated TOF, estimated from 5 independent replicas. All simulations were run for 2.5·10^8^ KMC steps, and the TOFs were computed only considering the second half of the simulated time, to account for the initial transient period. The remaining parameters were fixed to **DownLimit** = 5, *a*_3_ = 2 (for **MaxSep**) and *a*_4_ = 1 (for **UpLimit**).

The performance metrics are speedup and accuracy. Speedup is defined as the ratio of the mean number of KMC events needed to reach 0.4 ms of simulated time in nonaccelerated runs ⟨*N*_*t*=0.4ms_⟩_non–acc_ to that of the accelerated runs ⟨*N*_*t*=0.4 ms_⟩_acc_

25 $$\mathrm{speedup}=\frac{{\left\langle N_{t=0.4\mathrm{ms}}\right\rangle }_{\mathrm{non}‐\mathrm{acc}}}{{\left\langle N_{t=0.4\mathrm{ms}}\right\rangle }_{\mathrm{acc}}}$$

were ⟨·⟩ denotes the replica average. The value of 0.4 ms is chosen since it corresponds to approximately half of the total simulated time in the reference runs. The accuracy is quantified as the signed relative error in the average CO TOF: between the accelerated ⟨TOF_CO_⟩_acc_ and nonaccelerated ⟨TOF_CO_⟩_non–acc_ simulations

26 $$\varepsilon_{\mathrm{rel}}\left(\%\right)=\frac{{\left\langle {\mathrm{TOF}}_{\mathrm{CO}}\right\rangle }_{\mathrm{acc}}-{\left\langle {\mathrm{TOF}}_{\mathrm{CO}}\right\rangle }_{\mathrm{non}‐\mathrm{acc}}}{{\left\langle {\mathrm{TOF}}_{\mathrm{CO}}\right\rangle }_{\mathrm{non}‐\mathrm{acc}}}\times 100$$

where ⟨TOF_CO_⟩_acc_ and ⟨TOF_CO_⟩_non–acc_ are the mean TOFs for the accelerated and the nonaccelerated runs, respectively.

Figure 3 shows that most parameter sets keep the average TOF of the accelerated replicas within the ±2σ confidence band of the nonaccelerated simulations, while still achieving speedups of 4 – 10×. A few more aggressive combinations achieve speedups approaching 100×, but with *a* ∼ 40% error in the predicted TOF, a penalty that in many catalytic systems is still tolerable, given that small (Δ*E*_a_ ∼ 0.1 eV) errors in activation barriers of rate-limiting reactions can already change the TOF by a factor of exp­(Δ*E*_a_/*k*_B_ *T*) ≈ 5 at ∼700 K and 1 order of magnitude at ∼500 K.

The most influential parameter is the sampling window length, **CheckEvery** (Figure 3D, controlled via *a*_2_). The best performance is obtained for *a*_2_ = 5, where the algorithm exhibits speedups of 6 – 8× with negligible error, almost insensitive to the values of the other parameters. This value roughly matches the number of fast, quasi-equilibrated channels in the reaction model (six, see Figure 2D). The results for *a*_2_ = 2.5 also have negligible error but the speedup is lower (∼4×), since the short sampling windows prevent some fast channels from accumulating enough events to classify as *too fast* (Section 3.2). The results for *a*_2_ = 10 are the only ones that result in significant errors, although they can deliver speedups far greater than 10×.

Next in importance is **MinSep** (Figure 3A). As expected, the trend shows that increasing the time scale separation lowers both speedup and error. Values around 500–1000 offer a good compromise between speedup and accuracy, and values significantly larger than 1000 would result in negligible speedup. For **PEtol** the trend is also intuitive. A tight tolerance of 0.01 keeps errors low even when *a*_2_ = 10 but might become too strict for more complex reaction models with many active channels, since they lead to noisier statistics. A value of **PEtol** = 0.02 is more forgiving and, in this benchmark, is equally accurate provided the sampling window is not excessively long (*a*_2_ < 10). Finally, the effect of **PEminN** (Figure 3C, controlled via *a*_1_) is more subtle. Lowering *a*_1_ can raise the speedup without sacrificing accuracy, although this factor must remain greater than one to satisfy the constraint imposed by eq 20.

#### 4.2.2 Influence of Secondary Scaling Parameters

The impact of the three remaining parameters **DownLimit**, **MaxSep** and **UpLimit** has also been studied and is shown in Figures S1–S3. For each of these parameters we tested four representative values across all combinations of two representative levels for **MinSep** (500 and 1000), **PEtol** (0.01 and 0.02), **PEminN** (controlled by *a*_1_ values of 1.5 and 3) and **CheckEvery** (controlled by *a*_2_ values of 2.5 and 10). This yields 3 × 4 × 2^4^ = 192 distinct parameter sets for the accelerated simulations, and for each set we ran five statistically independent replicas to obtain averages.

**DownLimit** (Figure S1) spans from a very restrictive cap on how much channels can be adjusted after each sampling window (1.5) to essentially no cap (10^6^). No significant impact in speedup or accuracy is observed in any of the tested cases, apart from a slight decrease in speedup when the cap is decreased from 5 to 1.5. Because the intrinsic time scale separation of the RWGS model is modest (about 4 orders of magnitude, top panel in Figure 2D), fast channels still reach the target time scale window using a very restrictive cap relatively quickly, while using no cap (10^6^) is also safe because the statistics of channel frequencies in each sampling window are “clean” enough, implying that eqs 15–17 always provide good accurate target values. We recommend using **DownLimit** = 5 as a default value that provides a reasonable compromise between aggressiveness and caution. Only extremely stiff reaction models might require a higher value.

**MaxSep** (Figure S2, controlled via *a*_3_) defines the upper end of the permissible time scale window for quasi-equilibrated channels, but also affects the length of the sampling window over which statistics are collected (eq 22). This coupling leads to two different trends depending on the value of *a*_2_. When the sampling window is long (*a*_2_ = 10), increasing the upper end of the time scale window by raising *a*_3_ simply means that fewer channels are tagged as “too fast”, so the overall speedup decreases as expected. However, when the sampling window is short (*a*_2_ = 2.5), shrinking this window with a very small *a*_3_ (1.01, so **MaxSep** ≈ **MinSep**) simultaneously shortens **CheckEvery**, and the combined effect leads to noisy statistics that actually reduce the speedup. To avoid this coupling, we recommend keeping **MaxSep** = 2 × **MinSep** (*a*_3_ = 2) as a default and use the single factor *a*_2_ to tune the length of the sampling window when required.

Finally, **UpLimit** (Figure S3, controlled via *a*_4_) was varied from 0.3 (far lower upward than downward limit to changes in the stiffness coefficients) to 100 (far larger). We observe no significant changes in either speedup or accuracy across all parameter sets explored in this case. We recommend using *a*_4_ = 1 (**UpLimit** = **DownLimit**) to keep upscaling and downscaling adjustments symmetric.

### 4.3 Application to a Large and Stiff Model: DRM on Pt/HfC

We next analyze the performance of the algorithm on the DRM (CH_4_ + CO_2_ → 2CO + 2H_2_) on Pt/HfC model, previously studied by Prats et al.^9^ using a preliminary version of this algorithm. The model is used without modification and serves as a benchmark to test the performance of the algorithm in challenging KMC models, as it contains 775 lattice sites of three types (Figure 4C), a cluster expansion with 175 clusters, and a large and stiff reaction network with 119 reversible reaction channels whose time scales span many orders of magnitude. All details on this model are provided in ref 9. We explored 144 operating conditions on a 12 × 12 logarithmic grid in (*p*_CH_4__, *p*_CO_2__), covering 5 orders of magnitude in each partial pressure to test the algorithm performance under a broad range of surface coverages and kinetic regimes. We used the recommended parameter values, except for *a*_2_ (**CheckEvery**), which was increased from 5 to 30 due to the large number of fast channels in this model. We show later the impact of **MinSep** and *a*_2_ on the algorithm performance.

**Figure 4:**
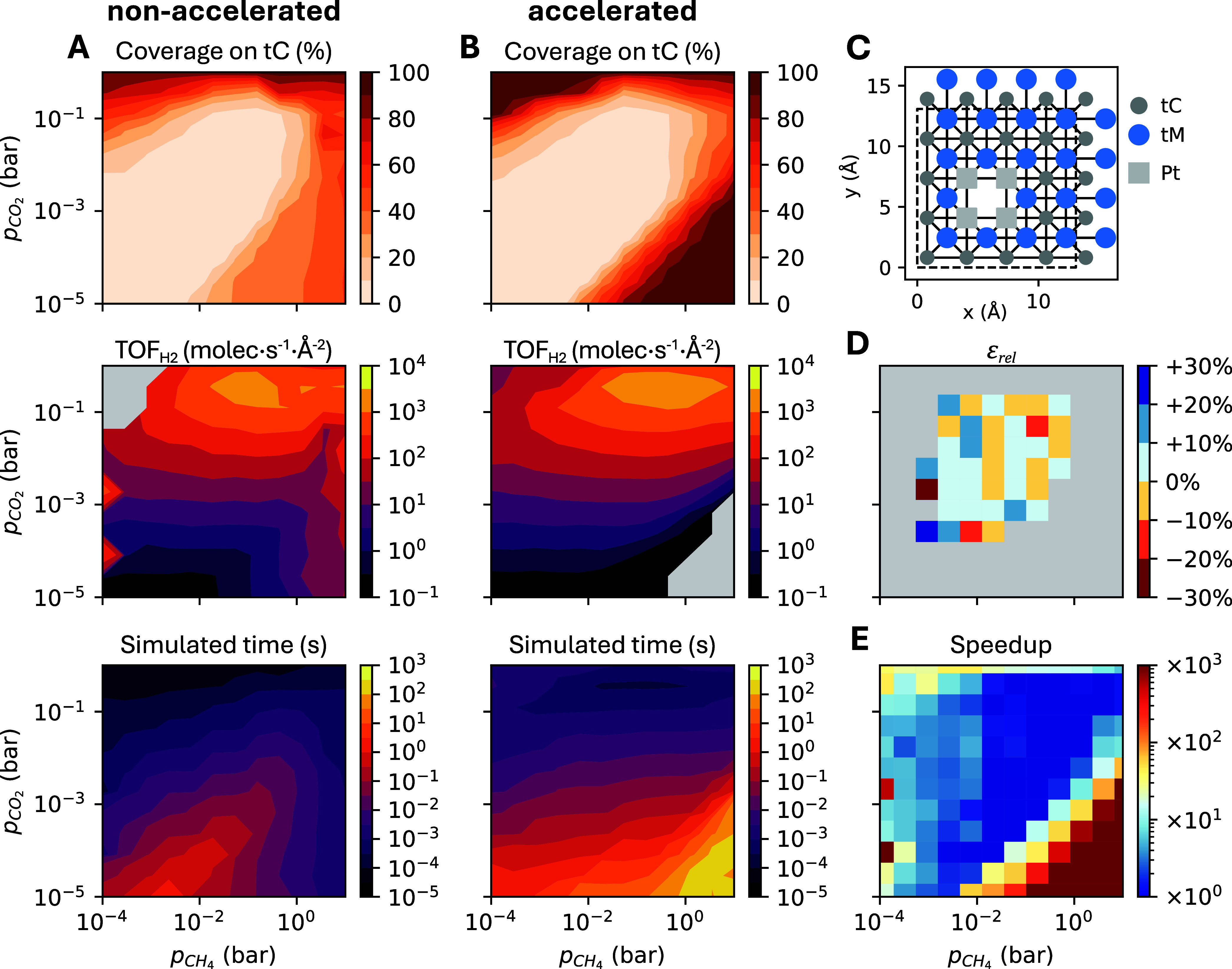
(A, B) Heatmaps of total surface coverage on tC sites (top), H_2_ TOF (middle) and total simulated time (bottom) for the nonaccelerated (A) and accelerated (B) simulations in the DRM on Pt/HfC model at 1100 K. In TOF heatmaps, gray areas indicate that the number of produced H_2_ molecules is lower than the threshold of 10 and therefore TOF is not computed. (C) Lattice model used for Pt/HfC. The unit cell is shown in dashed lines, while solid lines indicate site connectivity. (D) Signed relative error in the H_2_ TOF for the accelerated simulations, only calculated when the nonaccelerated results are converged and have a TOF ≥ 1.0 molec·s^–1^·Å^–2^. (E) Speedup achieved, calculated as the ratio of simulated times reached by the accelerated versus the corresponding nonaccelerated simulation. Fixed parameters: **PEtol** = 0.02, **MinSep** = 500, **DownLimit** = 5, *a*_1_ = 1.5 (**PEminN**), *a*_2_ = 30 (**CheckEvery**), *a*_3_ = 2 (**MaxSep**), and *a*_4_ = 1 (**UpLimit**). Each heatmap is based on a 12 × 12 logarithmically spaced grid of *p*_CH_4__ and *p*_CO_2__ conditions, ranging from 10^–4^–10^1^ bar for CH_4_ and 10^–5^–10^0^ bar for CO_2_. All simulations were run for 4·10^7^ KMC steps.

#### 4.3.1 Accuracy and Speedup across Operating Conditions

Without stiffness scaling, steady-state is reachable within a realistic time frame for only a limited number of operating conditions. Thus, we only quantify the accuracy of the scaling algorithm for these conditions using the same definition as in eq 26, but here using the signed relative error in the H_2_ TOF. To determine whether a nonaccelerated simulation has reached steady-state, we first ignore the first half of the total simulated time and, for the second half, we use a function implemented in the Python library ZacrosTools^35^ that checks whether (i) the slope of the surface energy vs number of KMC events is lower than a threshold, taken equal to 2·10^–10^ eV·Å^–2^·event^–1^, and (ii) that the simulated time vs number of KMC events curve is linear with *r* ^2^ > 0.95. The signed relative error is only computed for the conditions where both criteria are met. Since different operating conditions yield different simulated times, speedup is defined as the total simulated time for the accelerated simulation (*t*_acc_) divided by that of the nonaccelerated simulation (*t*_non–acc_) keeping the number of KMC steps constant, rather than via eq 25:

27 $$\mathrm{speedup}=\frac{t_{\mathrm{acc}}}{t_{\mathrm{non}‐\mathrm{acc}}}$$

Figure 4 compares results from nonaccelerated and accelerated simulations, presented as heatmaps of surface coverage, H_2_ TOF, and total simulated time. The TOF heatmap for the nonaccelerated simulations (Figure 4A) shows poor continuity, with fluctuations and singularities. The latter suggest that many simulations have still not reached stationarity. This effect is also visible—though less pronounced—in the surface coverage plot. These irregularities occur mainly at CH_4_ partial pressures above 1 bar, where multiple fast reaction and diffusion pathways involving CH_*x*_ species become active. In contrast, the accelerated simulations (Figure 4B) produce smooth, continuous heatmaps, indicating that the algorithm successfully accelerates all slow simulations across the entire range of operating conditions. Importantly, the acceleration remains controlled, with no signs of instability due to excessive upscaling or downscaling.

In conditions where the nonaccelerated simulations do reach steady-state (typically where surface coverage is low) and thus the error introduced by the stiffness scaling can be evaluated, Figure 4D shows that it is generally below 10%, with a few cases in the ±10–20% range, and only two conditions exceeding ±20%. The simulated time varies across several orders of magnitude depending on the operating conditions. The accelerated simulations progress much further, especially under high-coverage conditions. These conditions are typically characterized by long initial transient periods that, without stiffness scaling, would prevent the simulation from reaching stationarity in a realistic amount of time. As shown in Figure 4E, speedups of several orders of magnitude are achieved, especially in the bottom right corner of the plot. In Figure 4E, speedup values are capped at 1000× for clarity; higher values shown in red. However, a comparison of the simulated time heatmaps shows that speedups can reach up to 10^6^× at 10 bar CH_4_ and 10^–5^ bar CO_2_. Moderate to high speedups (10–100×) are also observed in other regions of high coverage. Conversely, in high-activity regions, speedups are low, as these conditions do not involve excessively fast channels and therefore require little to no downscaling. This confirms that the algorithm avoids unnecessary downscaling, even in complex models.

#### 4.3.2 Parameter Sensitivity in Large-Models

To evaluate the sensitivity of the algorithm to parameter selection, we focus on two key stiffness scaling parameters: **MinSep** and **CheckEvery** (via *a*_2_), which previously showed the strongest impact in the RWGS on Ni(111) model. We tested three representative values for each parameter, covering all 9 possible combinations. The results are very similar across them. The H_2_ TOF heatmaps (Figure 5A) exhibit identical volcano shapes with consistent trends; the peak appears at the same location in all cases. Only minor variations are observed in the top-left region of the plot. Similarly, the error heatmaps (Figure 5B) reveal no significant impact on accuracy, even when reducing **MinSep** to 200.

**Figure 5:**
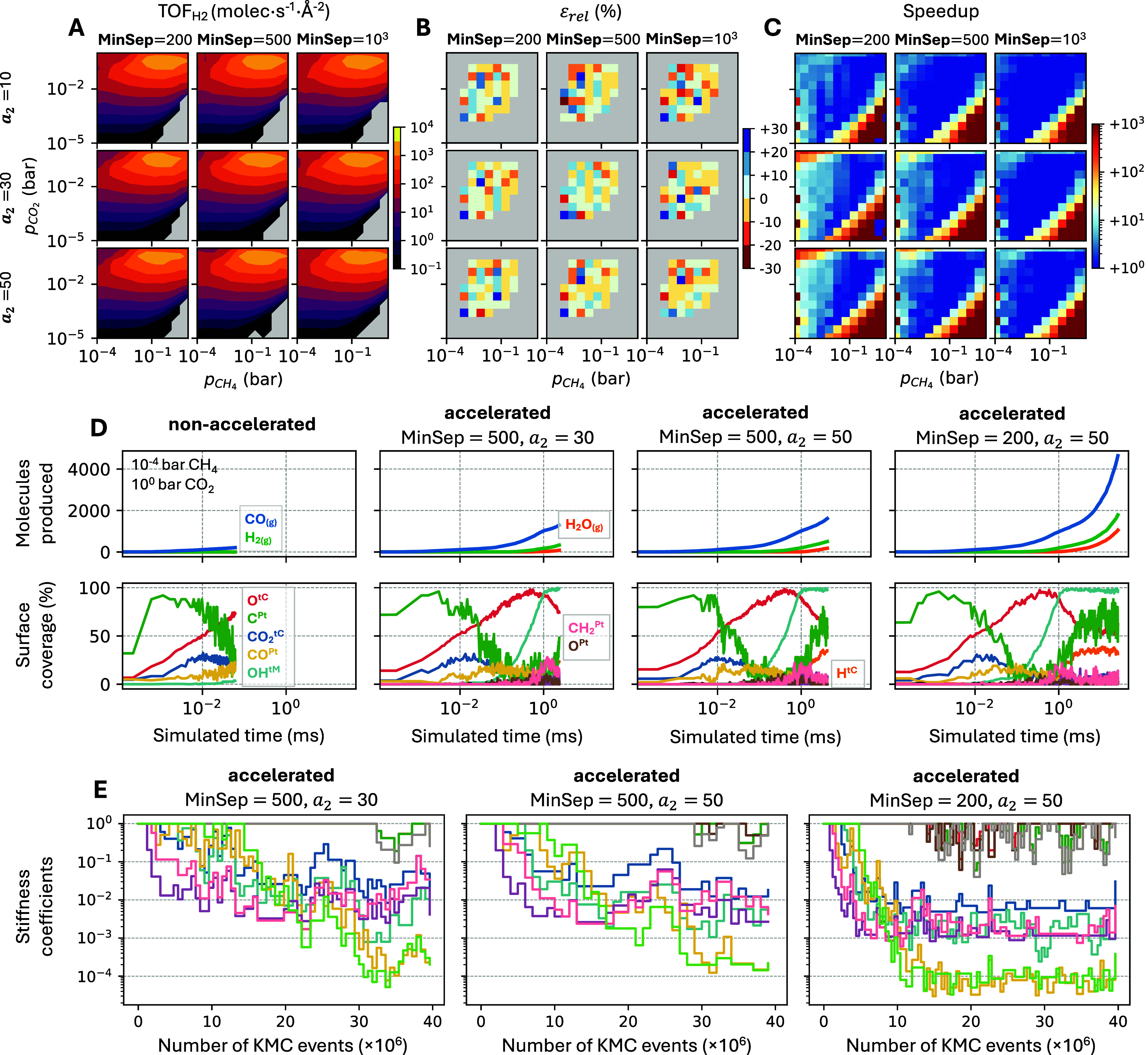
(A–C) Heatmaps for the DRM on Pt/HfC model at 1100 K, showing results from accelerated simulations for several combinations of **MinSep** and **CheckEvery** (via scaling factor *a*_2_). (A) H_2_ TOF; gray areas indicate conditions where fewer than 10 H_2_ molecules were produced, and TOF was not computed. (B) Signed relative error (%) in H_2_ TOF, only calculated when the nonaccelerated results are converged and have a TOF ≥ 1.0 molec·s^–1^·Å^–2^. (C) Achieved speedup, defined as the ratio of KMC steps in nonaccelerated versus accelerated simulations. Each heatmap is based on a 12 × 12 logarithmically spaced grid of *p*_*CH*_4__ and *p*_*CO*_2__ conditions, ranging from 10^–4^–10^1^ bar CH_4_ and 10^–5^–10^0^ bar CO_2_. (D) Cumulative gas-phase production (top) and coverage of surface species (bottom) at 10^–4^ bar CH_4_ and 10^0^ bar CO_2_ for the nonaccelerated case and three accelerated cases with different MinSep and *a*_2_ values. (E) Evolution of stiffness coefficients in the same accelerated runs shown in (D). The color legend mapping the stiffness coefficient curves of the downscaled channels to *Zacros*’s elementary channel names is provided in Figure S4 (SI). Other fixed parameters: **PEtol** = 0.02, **DownLimit** = 5, *a*_1_ = 1.5 (**PEminN**), *a*_3_ = 2 (**MaxSep**), and *a*_4_ = 1 (**UpLimit**). All simulations were run for 4·10^7^ KMC steps.

Differences among parameter combinations are most evident in the speedup maps (Figure 5C). As expected, speedup increases as **MinSep** decreases and *a*_2_ increases. Longer sampling windows reduce statistical noise in reaction occurrence counts, especially in models with many active reaction channels. With a longer window, fast channels fire enough times so that occurrence counts exceed the MaxDesiredN threshold, allowing these channels to be correctly identified for downscaling. The largest gains in speedup are observed in the top-left corner of the map. Decreasing **MinSep** from 500 to 200 and increasing *a*_2_ from 30 to 50 raises the speedup from approximately 10–50× to 100–1000×. Thus, while the recommended default parameters provide good performance across most of the explored conditions, further tuning of **MinSep** and **CheckEvery** may be required in specific, challenging regions.

A quantitative accuracy comparison is not possible in the conditions where the speedup is the highest, since the nonaccelerated simulation does not reach steady-state. Nevertheless, Figure 5D shows the evolution of cumulative product formation and surface coverages for the nonaccelerated simulation and three accelerated runs (each with different parameter combinations) at 10^–4^ bar CH_4_ and 1 bar CO_2_ (top-left corner of the grid). These are probably the most challenging conditions, as they involve prolonged initial transient periods and near-saturation of all three site types. Although stiffness scaling is applied from the beginning in the accelerated simulations (Figure 5E shows the stiffness scaling coefficients for these runs), they closely reproduce the early dynamics of the nonaccelerated simulation (rightmost 3 pairs of panels in Figure 5D). Up to the point where the nonaccelerated simulation terminates (∼0.06 ms), differences in the numbers of molecules produced and surface coverages are negligible. In contrast, the accelerated runs reach simulated times of 2, 4, and 26 ms within the same number of KMC steps. Since steady-state is reached only after ∼5 ms, only the fastest accelerated run yields converged results within the simulation window. This highlights the value of targeted parameter tuning in extreme cases. The simulation with **MinSep** = 200 and *a*_2_ = 50 is the most efficient in this specific case, achieving convergence faster by more aggressively downscaling fast channels. After ∼15 million KMC steps, the stiffness coefficients stabilize in this simulation, while in the other two simulations the time scales of fast channels are still being adjusting even at the end of the simulation. Nonetheless, such cases where additional tunning is needed are rare; across most of the parameter space, the default values suffice to achieve substantial speedups without compromising the accuracy.

### 4.4 Robustness under Transient Conditions: TPD on NiCu SAA

We now discuss benchmarks of the algorithm on a KMC model simulating the TPD spectra of formate dissociation to CO_2_ on a NiCu SAA, recently studied by Li et al.^36^ Here, we adopt a slightly modified version with fewer diffusion channels, allowing the nonaccelerated simulation to complete in a reasonable amount of (real) time. This system features transient kinetics under a temperature ramp, where certain channels begin as fast but eventually slow down.

#### 4.4.1 Mechanistic Description and Kinetic Regimes

A key feature of the model is that formate (HCOO) species are treated as bidentate: they can occupy either two Cu sites (HCOO^Cu/Cu^) or a Cu site and a dopant Ni site (HCOO^Cu/Ni^). Since the simulation distinguishes the two different configurations, each “main” reaction channel (e.g., diffusion of HCOO on Cu) is split into several independent channels to account for the different orientations of the adsorbates.

The simulation is initialized with 1000 HCOO^Cu/Cu^ species adsorbed on a 20,000-site lattice, with a Ni loading of 0.7%. At the start, the temperature (100 K) is too low for HCOO dissociation to occur, so only diffusion channels involving HCOO on Cu are active. As the simulation progresses and temperature increases, HCOO species begin populating Ni sites (Figure 6A), activating additional diffusion channels involving Ni. Around 200–250 K, the first HCOO molecules begin to dissociate near Ni sites. This dissociation channel remains nonequilibrated and only occurs in the forward direction. Simultaneously, the overall rate of H diffusion on Cu increases rapidly, due to the increased number of H* adatoms on the surface. By 250 K, nearly all Ni dopant sites are saturated with HCOO (Figure 6A). At around 300 K, HCOO dissociation becomes fast on both Cu and Ni, producing CO_2(g)_ and additional surface H atoms. The H species subsequently diffuse and recombine to form H_2(g)_ (Figure 6B). Most HCOO species dissociate on Ni sites, where the barrier is lower. As HCOO coverage decreases, its diffusion channels begin to slow down. Approaching the CO_2_ desorption peak at 312 K (Figure 6C), H coverage becomes negligible, and H diffusion also slows down. Shortly after the peak, all species have desorbed, no further reactions occur, and the simulation terminates.

**Figure 6:**
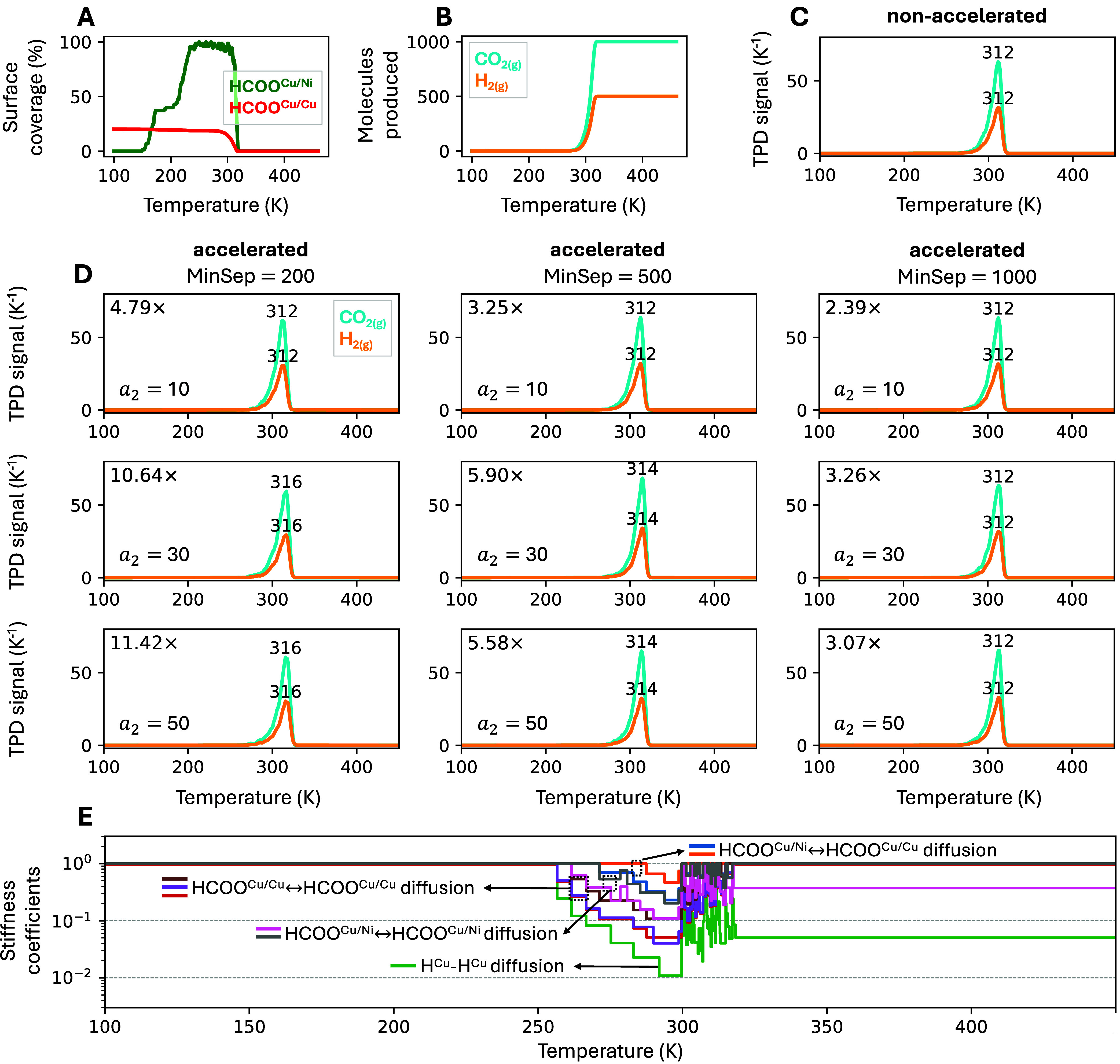
Results for the TPD simulations of formate dissociation on NiCu SAA. A temperature ramp was specified in the simulation, with an initial temperature of 100 K and a rate of change of 2 K per second. The partial pressures for all gas-phase species were set to zero. (A, B) Surface coverage of HCOO (A) and cumulative gas-phase production of CO_2_ and H_2_ (B) as a function of simulated time for a representative nonaccelerated replica. The coverage of H remains below 1% and is not shown. (C–D) Simulated TPD spectra obtained from nonaccelerated (C) and accelerated (D) simulations for various combinations of **MinSep** and **CheckEvery** (via *a*_2_). Each spectrum is obtained by averaging over four independent replicas with different random seeds. The peak positions and speedup in each accelerated simulation are labeled. (E) Evolution of stiffness coefficients vs temperature for one representative replica of the accelerated simulation with **MinSep** = 500 and *a*_2_ = 10. Fixed parameters: **PEtol** = 0.02, **DownLimit** = 5, *a*_1_ = 1.5 (**PEminN**), *a*_3_ = 2 (**MaxSep**), and *a*_4_ = 1 (**UpLimit**).

#### 4.4.2 Accuracy and Speedup in TPD Simulations

The nonaccelerated simulation shows desorption peaks at 312 K for both CO_2_ and H_2_ (Figure 6C) and requires approximately 6·10^7^ KMC events to complete. In contrast, the accelerated simulations yield peak positions between 312 and 316 K (Figure 6D), depending on the parameters used. The fastest case (**MinSep** = 200 and *a*_2_ = 50) completes the simulation in only ∼5·10^6^ KMC events, a speedup of ∼11×. Here, speedup is computed analogously to eq 25:

28 $$\mathrm{speedup}=\frac{{\left\langle N\right\rangle }_{\mathrm{non}‐\mathrm{acc}}}{{\left\langle N\right\rangle }_{\mathrm{acc}}}$$

where *N* is the number of KMC events required to complete the simulation and ⟨·⟩ denotes the replica average.

When *a*_2_ = 10, which is approximately equal to the number of fast channels (eight), no loss in accuracy is observed, despite achieving a speedup of up to ∼5× (for **MinSep** = 200). Increasing *a*_2_ to higher values (30 and 50) further increases the speedup but introduces small errors, except in the case of **MinSep** = 1000, where accuracy is preserved, though speedup gains are minimal. This behavior likely originates from the reduced frequency of stiffness scaling updates at high *a*_2_, which makes the algorithm less responsive to rapidly evolving kinetics. Despite this, the algorithm adapts well to transient regimes, as illustrated by the evolution of stiffness coefficients in Figure 6E. Initially, the coefficients for fast, quasi-equilibrated channels are progressively downscaled, followed by a sharp upscaling phase once HCOO is depleted. In other TPD models in which stiffness is more pronounced, a higher **UpLimit** value might be beneficial to accelerate the recovery of downscaled coefficients to their original rates. However, in this case, the default value of 5 (corresponding to *a*_4_ = 1) is shown to be sufficient.

## 5 Potential Limitations

The algorithm is highly flexible, featuring seven user-adjustable parameters that allow fine control over its behavior. However, this flexibility can also introduce complexity, particularly for nonexpert users. To address this, the preceding sections demonstrate that the recommended default settings provide robust performance across a wide range of systems. The only parameter for which a reliable default is more difficult to provide is the sampling window length, **CheckEvery** (controlled via the scaling factor *a*_2_). Our results indicate that the optimal performance is achieved with a value of *a*_2_ similar to the number of fast, quasi-equilibrated channels in the system, information that is generally not known a priori, especially in complex or heterogeneous KMC models. As a result, users may need to experiment with different values to find a suitable setting. Future versions of the algorithm could address this by implementing an adaptive sampling window length, dynamically adjusted during the simulation based on the number of active fast channels detected on-the-fly.

## 6 Implementation in *Zacros*

The algorithm has been implemented in the *Zacros* code and will be included in the upcoming *Zacros* 5.0 release. The implementation closely follows the workflow outlined in Figure 1, with two technical considerations specific to *Zacros*. First, *Zacros* allows users to specify which reaction channels are eligible for stiffness scaling using the stiffness_scalable and stiffness_scalable_symmetric keywords within each reaction block in the mechanism_input.dat file. Channels that do not include either keyword are treated as nonscalable and are excluded from any downscaling operations. This feature could be useful in complex KMC models, where the user may wish to exempt known rate-limiting channels from downscaling to prevent distorting the simulated dynamics. Nonetheless, in all case studies presented in this work including the large DRM model with 119 reaction channels, all channels were treated as stiffness-scalable, and the algorithm autonomously determined which channels to scale and when. Second, *Zacros* supports the definition of irreversible reaction channels. These channels are automatically classified as nonequilibrated, and no PE ratio is calculated for them, which avoids unnecessary computations.

## 7 Conclusions

We have developed a general and robust stiffness-scaling algorithm that enables efficient and accurate acceleration of kinetic Monte Carlo (KMC) simulations of catalytic systems with complex reaction networks. The method dynamically detects fast, quasi-equilibrated channels based on event statistics and adjusts their rate constants through controlled upscaling and downscaling, overcoming key limitations of earlier strategies.

The algorithm was systematically validated across three representative models: the RWGS reaction on Ni(111), the DRM on Pt/HfC, and a TPD simulation of formate dissociation on NiCu SAAs. In all cases, it achieved significant speedups, reaching up to several orders of magnitude in highly stiff regimes, while maintaining high accuracy in predicted observables. Sensitivity analyses revealed that the algorithm is largely robust to parameter choices, with only the sampling window length (**CheckEvery**) requiring tuning in certain scenarios. The algorithm performs reliably even in challenging conditions, such as large and stiff reaction networks with more than 100 reaction channels, or transient kinetics, and avoids unnecessary scaling in regimes where it is not beneficial. It has been fully implemented in the *Zacros* software package and is compatible with realistic surface models featuring lateral interactions and multiple site types.

We provide default parameter values that offer good performance across a wide range of systems, while allowing expert users to further fine-tune behavior when needed. Overall, this stiffness-scaling framework significantly extends the applicability of KMC simulations in heterogeneous catalysis and materials science by enabling tractable simulations of complex systems that were previously prohibitively expensive.

## Supplementary Material



## Data Availability

*Zacros* input files for KMC simulations as well as Python scripts to prepare them and plot the results will be made available on Zenodo (DOI: 10.5281/zenodo.16790482) upon publication.

## Appendix A: Proof of Equation 20

From the quasi-equilibration condition

$$\left|\frac{N_{\mathrm{fwd}}}{N_{\mathrm{fwd}}+N_{\mathrm{rev}}}-\frac{1}{2}\right|<\mathrm{PEtol}$$

$$\Leftrightarrow \frac{\left|N_{\mathrm{fwd}}-N_{\mathrm{rev}}\right|}{N_{\mathrm{fwd}}+N_{\mathrm{rev}}}<2\mathrm{PEtol}$$

If *N*_fwd_ ≠ *N*_rev_

$$\Leftrightarrow \frac{N_{\mathrm{fwd}}+N_{\mathrm{rev}}}{\left|N_{\mathrm{fwd}}-N_{\mathrm{rev}}\right|}>\frac{1}{2\mathrm{PEtol}}$$

Use the identity

$$\max\left(a,b\right)=\frac{a+b+\left|a-b\right|}{2}\;with\;a=N_{fwd},b=N_{rev}$$

$$\max\left(N_{\mathrm{fwd}},N_{\mathrm{rev}}\right)=\frac{N_{\mathrm{fwd}}+N_{\mathrm{rev}}+\left|N_{\mathrm{fwd}}-N_{\mathrm{rev}}\right|}{2}$$

$$\Leftrightarrow N_{\mathrm{fwd}}+N_{\mathrm{rev}}=2\max\left(N_{\mathrm{fwd}},N_{\mathrm{rev}}\right)-\left|N_{\mathrm{fwd}}-N_{\mathrm{rev}}\right|$$

Inserting this result into the inequality

$$\frac{2\max\left(N_{\mathrm{fwd}},N_{\mathrm{rev}}\right)-\left|N_{\mathrm{fwd}}-N_{\mathrm{rev}}\right|}{\left|N_{\mathrm{fwd}}-N_{\mathrm{rev}}\right|}>\frac{1}{2\mathrm{PEtol}}$$

$$\Leftrightarrow \frac{2\max\left(N_{\mathrm{fwd}},N_{\mathrm{rev}}\right)}{\left|N_{\mathrm{fwd}}-N_{\mathrm{rev}}\right|}>\frac{1}{2\mathrm{PEtol}}+1$$

$$\Leftrightarrow \max\left(N_{\mathrm{fwd}},N_{\mathrm{rev}}\right)>\frac{\mathrm{PEtol}+\frac{1}{2}}{2\mathrm{PEtol}}\left|N_{\mathrm{fwd}}-N_{\mathrm{rev}}\right|$$

For the case |*N*_fwd_ – *N*_rev_| = 1

$$\max\left(N_{\mathrm{fwd}},N_{\mathrm{rev}}\right)>\frac{\mathrm{PEtol}+\frac{1}{2}}{2\mathrm{PEtol}}$$

## Notes

Additional simulation data and methodological details; influence of **DownLimit** on speedup and accuracy (Figure S1); influence of *a*_3_ (**MaxSep**) on speedup and accuracy (Figure S2); influence of *a*_4_ (**UpLimit**) on speedup and accuracy (Figure S3); mapping of stiffness coefficients to *Zacros* step names and color legend for Figure 5E (Figure S4) (PDF)

## †

Institute of Materials Chemistry, Technische Universität Wien, 1060 Vienna, Austria
