# Supplementary material for: A General Stiffness-Scaling Framework for Accelerating Graph-Theoretical Kinetic Monte Carlo Simulations
Source: J Chem Theory Comput. 2025 Nov 18;21(23):12262–77. doi: 10.1021/acs.jctc.5c01394 (PMC12874366; doi:10.1021/acs.jctc.5c01394)

A General Stiffness-Scaling Framework for Accelerating Graph-Theoretical Kinetic Monte Carlo Simulations

Hector Prats*[a,b], Weitian Li[a] and Michail Stamatakis*[a]

[a] *Department of Chemistry, Inorganic Chemistry Laboratory, University of Oxford, South Parks Road, Oxford OX1 3QR, U.K.*

[b] *Current address: Institute of Materials Chemistry, Technische Universität Wien, 1060 Vienna, Austria*

*Corresponding authors: Hector Prats (hector.prats@tuwien.ac.at),

Michail Stamatakis (michail.stamatakis@chem.ox.ac.uk)

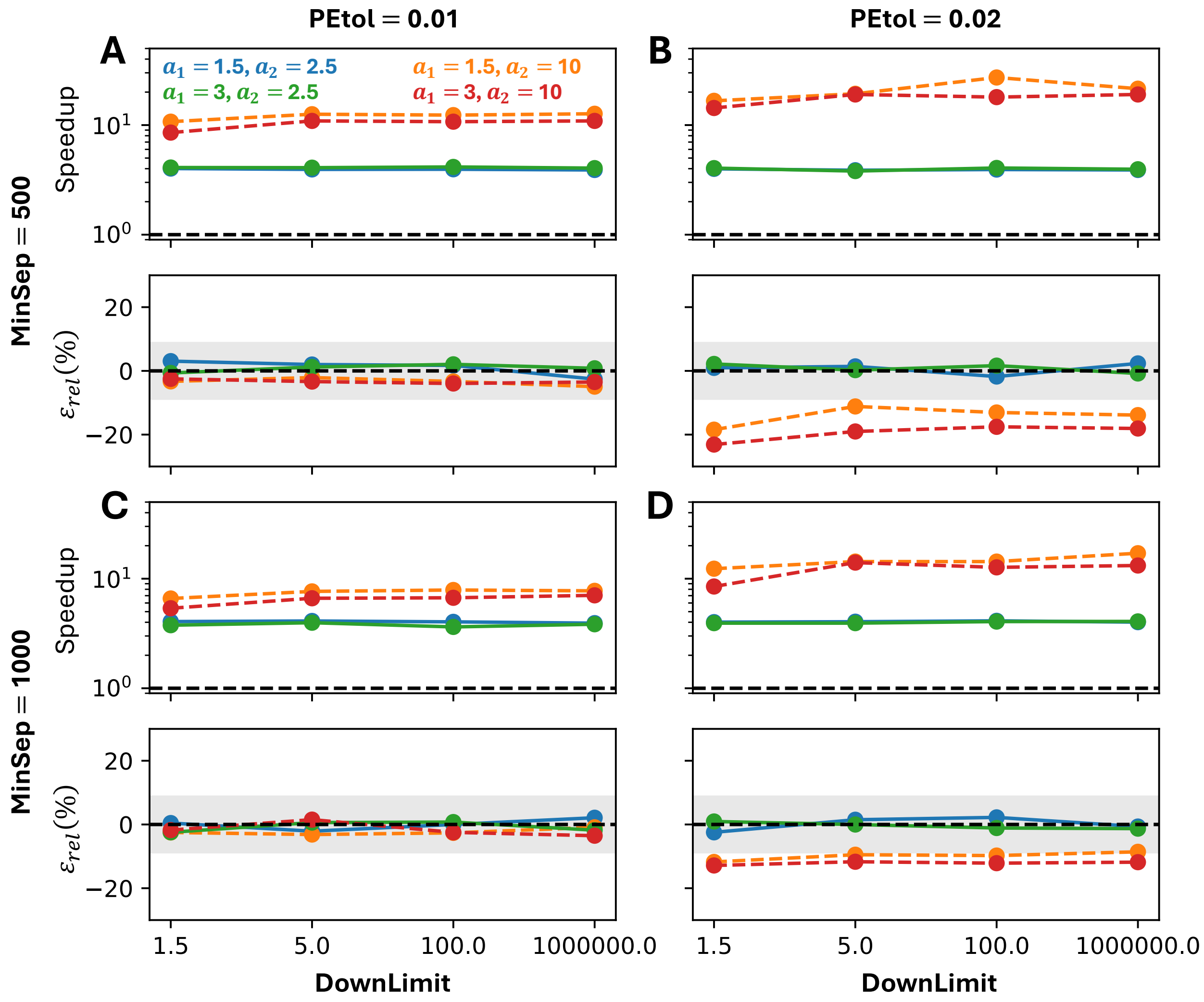

Figure S1. Influence of **DownLimit** on speedup and signed relative error in CO TOF for the accelerated algorithm on the RWGS on Ni(111) model (800 K, p_{CO2}=0.4 bar, and p_{H2}=1.6 bar). **MinSep** is fixed to 500 in the top row (A, B) and 1000 in the bottom row (C, D). The left and right columns use **PEtol**=0.01 and **PEtol**=0.02, respectively. Within each panel, four colour-coded curves correspond to the four combinations explored for scaling factors a_1 (for **PEminN**) and a_2 (for **CheckEvery**). The remaining parameters were set to $a_3 = 2$ (for **MaxSep**) and $a_4 = 1$ (for **UpLimit**). The speedup and signed relative error $\varepsilon_{rel}(\%)$ are defined according to Eqs. 25 and 26. Each data point is obtained by averaging over five independent replicas with different random seeds. The light-grey horizontal band indicates $\pm 2\sigma$ of the non-accelerated TOF, estimated from 5 independent replicas. All simulations were run for $2.5 \cdot 10^8$ KMC steps, and the TOFs were computed only considering the second half of the simulated time, to account for the initial equilibration period.

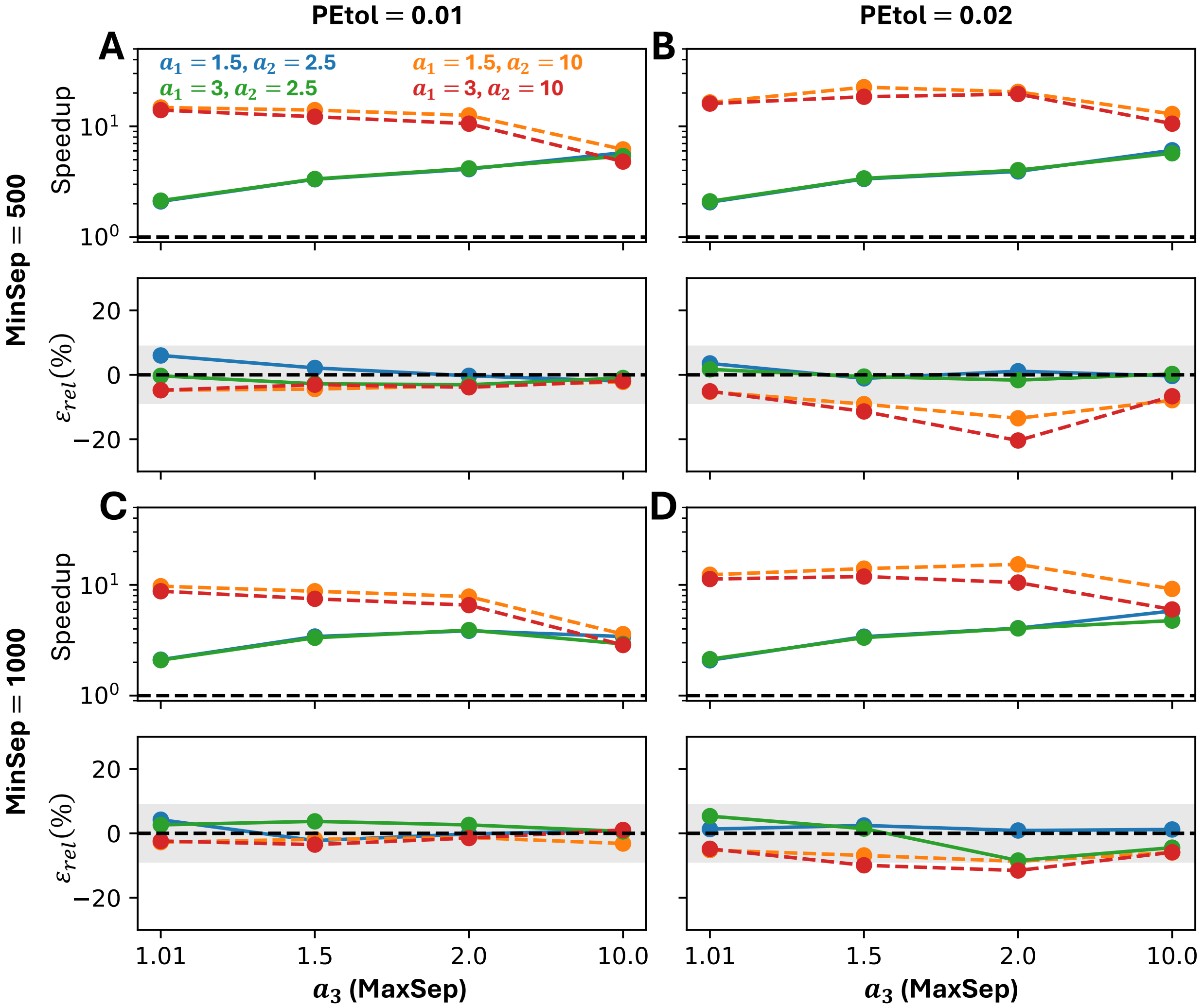

Figure S2. Influence of a_3 (for **MaxSep)** on speedup and signed relative error in CO TOF for the accelerated algorithm on the RWGS on Ni(111) model (800 K, p_{CO2}=0.4 bar, and p_{H2}=1.6 bar). **MinSep** is fixed to 500 in the top row (A, B) and 1000 in the bottom row (C, D). The left and right columns use **PEtol**=0.01 and **PEtol**=0.02, respectively. Within each panel, four colour-coded curves correspond to the four combinations explored for scaling factors a_1 (for **PEminN**) and a_2 (for **CheckEvery**). The remaining parameters were set to **DownLimit**=5 and $a_4 = 1$ (for **UpLimit**). The speedup and signed relative error $\varepsilon_{rel}(\%)$ are defined according to Eqs. 25 and 26. Each data point is obtained by averaging over five independent replicas with different random seeds. The light-grey horizontal band indicates $\pm 2\sigma$ of the non-accelerated TOF, estimated from 5 independent replicas. All simulations were run for $2.5 \cdot 10^8$ KMC steps, and the TOFs were computed only considering the second half of the simulated time, to account for the initial equilibration period.

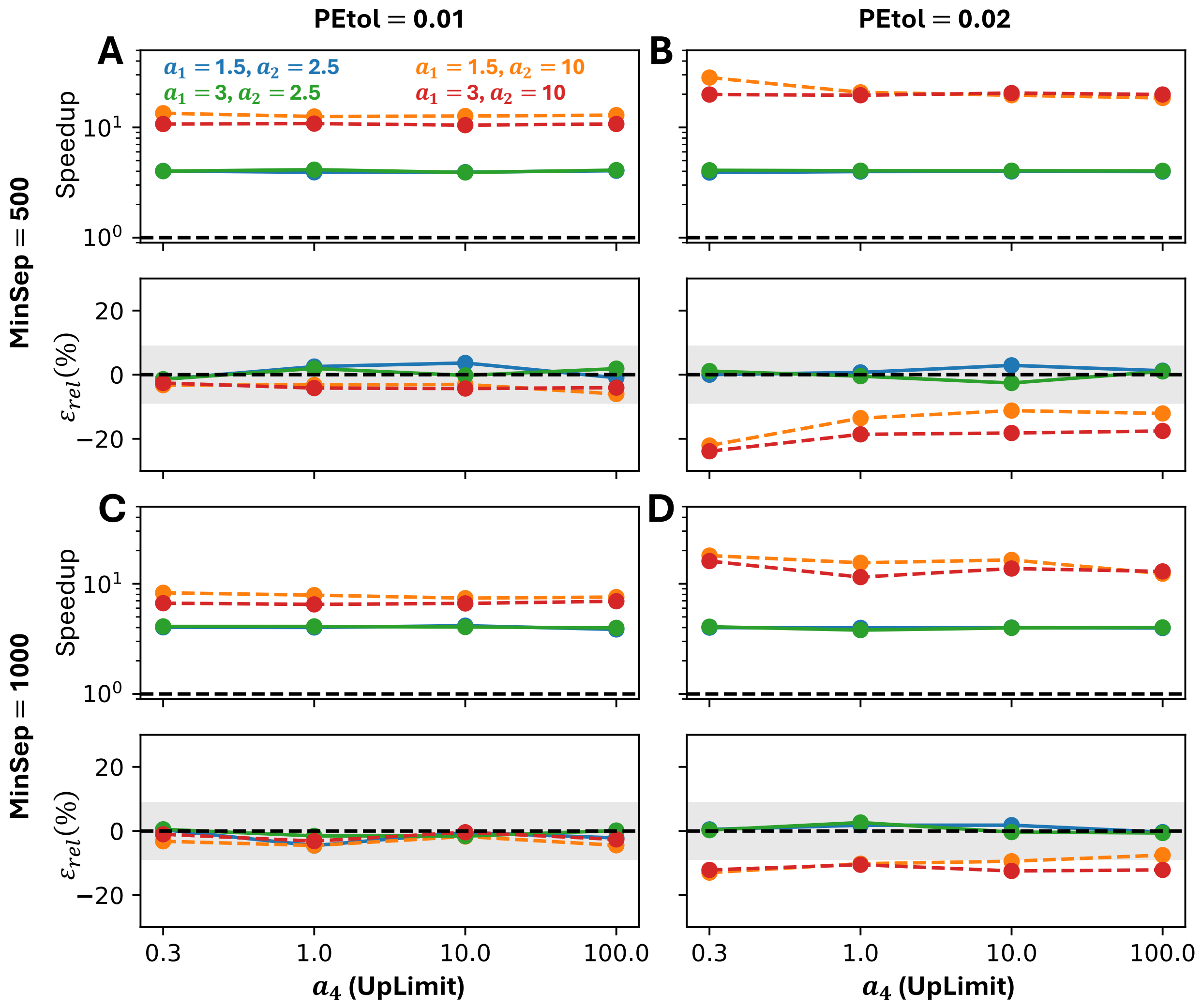

Figure S3. Influence of a_4 (for **UpLimit)** on speedup and signed relative error in CO TOF for the accelerated algorithm on the RWGS on Ni(111) model (800 K, p_{CO2}=0.4 bar, and p_{H2}=1.6 bar). **MinSep** is fixed to 500 in the top row (A, B) and 1000 in the bottom row (C, D). The left and right columns use **PEtol**=0.01 and **PEtol**=0.02, respectively. Within each panel, four colour-coded curves correspond to the four combinations explored for scaling factors a_1 (for **PEminN**) and a_2 (for **CheckEvery**). The remaining parameters were set to **DownLimit**=5 and $a_3 = 2$ (for **MaxSep**). The speedup and signed relative error $\varepsilon_{rel}(\%)$ are defined according to Eqs. 25 and 26. Each data point is obtained by averaging over five independent replicas with different random seeds. The light-grey horizontal band indicates $\pm 2\sigma$ of the non-accelerated TOF, estimated from 5 independent replicas. All simulations were run for $2.5 \cdot 10^8$ KMC steps, and the TOFs were computed only considering the second half of the simulated time, to account for the initial equilibration period.

Figure S4. Figure 5E in the main text including the color legend, which maps the stiffness coefficient curves of the downscaled channels to Zacros's elementary step names. Full channel definitions are provided in the accompanying Zenodo dataset (DOI: 10.5281/zenodo.16790482). See `system_2_drm_pthfc/csv_files/mechanism_data.csv` and the Zacros input/output files under `system_2_drm_pthfc/accelerated` and `system_2_drm_pthfc/non_accelerated`. Conditions and fixed parameters are as in Figure 5.

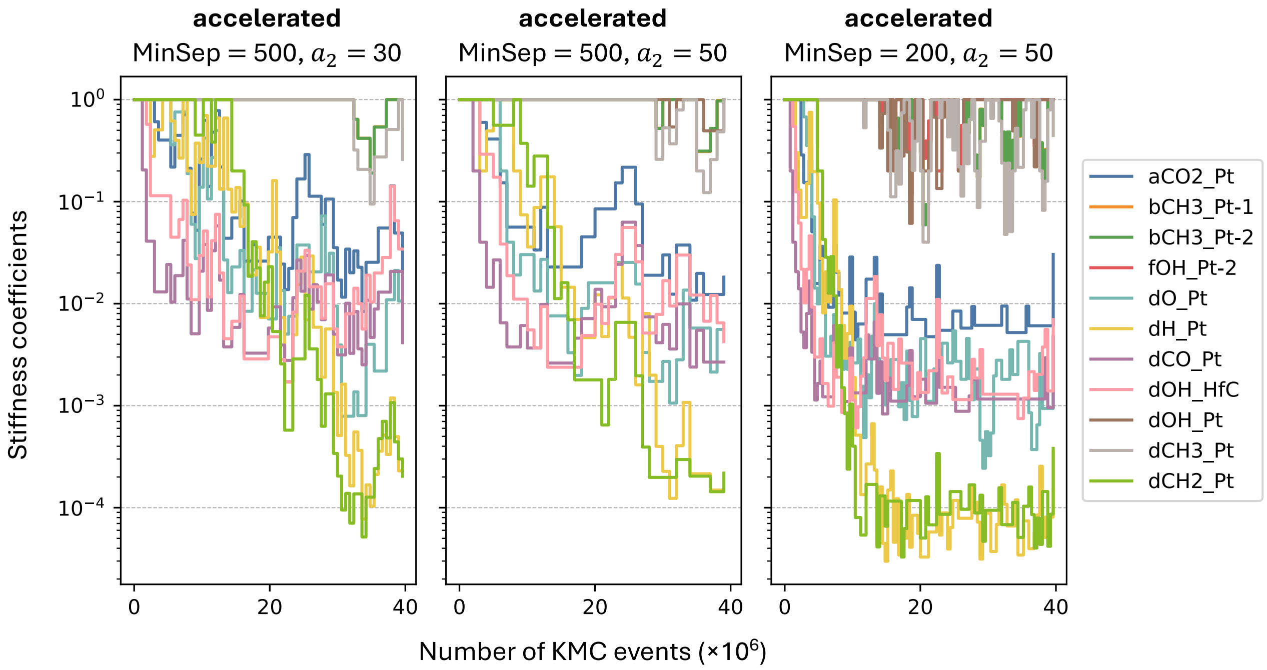

Supplement: Supplementary file 1 [file ct5c01394_si_001.pdf]
